# Voluntary sector specialist service provision and commissioning for victim-survivors of sexual violence: results from two national surveys in England

**DOI:** 10.1136/bmjopen-2024-087810

**Published:** 2024-09-13

**Authors:** Sarah Damery, Clare Gunby, Lucy Hebberts, Laura Patterson, Harriet Smailes, Jenny Harlock, Louise Isham, Fay Maxted, Jason Schaub, Deb Smith, Julie Taylor, Caroline Bradbury-Jones

**Affiliations:** 1 Applied Health Sciences, University of Birmingham, Birmingham, UK; 2 Manchester Metropolitan University, Manchester, UK; 3 Warwick Medical School, University of Warwick, Coventry, UK; 4 Department of Social Work and Social Care, University of Birmingham, Birmingham, UK; 5 The Survivors' Trust, Rugby, UK; 6 School of Nursing and Midwifery, University of Birmingham, Birmingham, UK

**Keywords:** Surveys and Questionnaires, Health Services, SEXUAL MEDICINE

## Abstract

**Abstract:**

**Background:**

In England, voluntary sector specialist (VSS) services are central to supporting victim-survivors of sexual violence (SV). However, empirical evidence is lacking about the scope, range and effectiveness of VSS provision for SV in England.

**Objectives:**

To undertake national surveys to map SV VSS service provision and describe arrangements for funding and commissioning.

**Design:**

Cross-sectional surveys.

**Setting:**

VSS services for SV and commissioners from multiple organisations across England (January–June 2021).

**Methods:**

Senior staff working in VSS services and commissioners from multiple organisations were surveyed electronically. Surveys explored SV service commissioning, funding and delivery, partnerships between organisations, perceived unmet need for services, and views about facilitators and challenges. Data were analysed descriptively to characterise VSS service provision for SV and commissioning across England.

**Results:**

54 responses were received from VSS providers and 34 from commissioners. Data demonstrated a complex and evolving funding and commissioning landscape in which providers typically secured funding from multiple sources, impacting consistency and scope of service provision. It was common for multiple organisations to co-commission services, demonstrating trends towards larger contracts that may disadvantage smaller specialist providers. Numerous examples of partnership working between organisations were identified, although developing partnerships was noted as challenging, particularly between VSS organisations. There was clear evidence of unmet need for services, with some groups of victim-survivors such as those from black and minority ethnic groups, often underserved by specialist services. However, there was also evidence of innovative service development and commissioning approaches to meet the needs of victim-survivors who face challenges accessing services.

**Conclusions:**

This study provides novel insights into SV service provision and commissioning in England, including unmet needs among victim-survivors.

**STRENGTHS AND LIMITATIONS OF THIS STUDY**

- This empirical study focuses specifically on voluntary sector specialist service provision and commissioning for sexual violence in England using a comparative methodological approach to understanding provider and commissioner perspectives.
- Data were collected using non-validated surveys developed following the literature review and after qualitative research to define key areas of questioning.
- The number of responses was comparatively small so conclusions cannot be drawn for the entire service provision and commissioning sector across England.
- Due to our pragmatic approach to survey dissemination to maximise participation, response rates cannot be calculated as the number of recipients is unknown.

## Introduction

 In 2021, there were an estimated 164 000 voluntary sector organisations in the UK.^1^ Also known as ‘third sector’ organisations, these bodies are independent of local and national government and have numerous attributes distinguishing them from statutory or private sector organisations.^2^ For example, they may be organised at a local or national level and use volunteers or a mix of volunteers and paid staff, depending on organisation size and scope.^3^ They are typically values-led and obtain funding from a range of sources including public donations, grants or contracts for delivering services. They have been described as having flat organisational hierarchies which lessen the distinction between staff, volunteers and service users,^4 5^ and service users are often involved in organisational governance. Voluntary sector services are fundamentally important in supporting people who may fall outside of other public sector support.^3^ Many voluntary sector bodies are grassroots organisations developed in response to local needs.^4^ The broader voluntary sector encompasses voluntary sector specialist (VSS) services, which provide specialist support such as counselling, therapy, advice, signposting to other services or advocacy for service users with specific needs.

There has been increasing recognition in England that VSS services are central to providing crisis and ongoing support to victim-survivors of sexual violence (SV).^6^,^8^ SV, defined as ‘any sexual act or attempted sexual act that takes place without consent’,^9^ was reported by 1.1 million people aged over 16 in the year ending March 2022 in England (a prevalence of at least 3.3% of women and 1.2% of men).^10^ In 2018, it was estimated that there were at least 207 VSS organisations providing services specific to SV in England,^11^ ranging in size, scope and geographical reach. Such organisations often incorporate specialised services such as independent sexual violence advisors (ISVA) (providing specialist-tailored practical/emotional support) and operate alongside clinical, mental health and social care providers. They can be accessed through diverse pathways, including self-referral, referral from health and social care or via the Police or Sexual Assault Referral Centres (SARCs).^12^ Many VSS services for SV support users from specific groups, such as children and young people (CYP), men or people from specific ethnic backgrounds, and the majority are affiliated to at least one national umbrella organisation such as The Survivors Trust, Rape Crisis or the Male Survivors Partnership.^11^

Despite their importance in providing for victim-survivors’ needs,^13^ funding for SV services has become increasingly complex over the last decade.^14^ Most VSS services derive funding from multiple sources, including charitable trusts and local/national statutory sources, disbursed via health (eg, Integrated Care Boards (ICB), National Health Service (NHS) England), local authorities, the Office of the Police and Crime Commissioner (OPCC) and criminal justice organisations. Service commissioning (ie, the planning and resourcing of services through different funding mechanisms in response to need) is also complex,^15 16^ with a trend towards larger contracts fulfilled by consortia and multidisciplinary partnerships potentially excluding smaller, locally specialised organisations from bidding to provide services.^17 18^ Consequently, austerity-driven funding cuts and changes to structure and funding of health and criminal justice services have led to gaps in service provision across England despite increasing demand.^19^ Resource limitations act as both organisational and systemic barriers, with direct impacts on the numbers of skilled staff within both the statutory and non-statutory sectors. Numerous studies have described the challenges posed by insufficient funding for SV support and in particular impacts on providers’ ability to deliver the appropriate level of specialised support to victim-survivors of SV.^20 21^ While in some areas, new models of collaborative commissioning have developed,^22 23^ alongside evidence of VSS organisations developing new collaborations and partnerships,^24^ little is known about the way that these changes have impacted on the services available to victim-survivors and their ability to access them.

Small-scale studies suggest that the independence of VSS services from statutory provision,^25^ their flexibility,^26^ local focus and potential for providing long-term rather than time-limited specialist support^27^ are key determinants of users’ satisfaction.^28^ However, there is also evidence of geographical variation in the level and types of services offered and under-representation of specific groups of victim-survivors in services.^29^,^31^ There may also be inconsistencies in the ways that VSS services identify and engage with underserved populations, which may impact the quality and effectiveness of support that service users receive.^32^ The PROSPER study^33^ (online supplemental file 1), funded by the UK National Institute for Health and Care Research (NIHR), used a multimethod, coresearch design to develop a national profile of VSS services in England and make recommendations about service provision for victim-survivors of SV. One component of the study was to survey senior staff working in VSS services and commissioners from local authorities, Clinical Commissioning Groups (CCGs), OPCC, the NHS, and health and justice organisations. Surveys aimed to ‘map’ VSS service provision for SV across England and describe service funding and commissioning to identify key trends and issues.

## Methods

Surveys were cross-sectional, developed following a literature/policy review and using qualitative data collected from service providers, commissioners, policy leads and victim-survivors of SV in the initial phase of PROSPER. This defined the broad areas covered, such as features of service organisation, services offered, contracting, funding and partnership. Survey methods are reported using the CROSS checklist^34^ (online supplemental file 2).

### Survey design and administration

Surveys were disseminated in parallel using the JISC Online Survey tool,^35^ between 13 January 2021 and 20 June 2021. The VSS provider survey (online supplemental file 3) comprised 64 questions (across 11 sections); the commissioner survey (online supplemental file 4) contained 51 (10 sections), and each took up to 25 min to complete. Surveys explored key aspects of SV service commissioning, funding and delivery, including what services are commissioned/provided, how and to which groups of victim-survivors; relationships and partnerships between organisations; sources of funding and trends over time, and commissioning approaches/models, including perceived effectiveness, facilitators and challenges. Surveys were designed to enable comparisons between the two participant groups on a number of important themes (eg, views about funding and commissioning, perceived strengths of specialist SV services, and under-representation of specific groups of victim-survivors in services). Survey questions required dichotomous answers, selection of one or more options from a list or 5-point Likert scale responses assessing respondent agreement with statements about specific aspects of services or commissioning. Respondents could also provide additional detail using free text. Draft surveys were piloted for relevance and readability by VSS service staff (provider survey) and commissioners (commissioner survey) within the West Midlands.

### Eligibility and recruitment

Potential participants (table 1) accessed surveys via a weblink embedded within a brief invitation email. The research team sent invitations directly to named VSS providers and commissioner contacts, for onward dissemination to the most relevant person within each organisation. Weblinks were also circulated by members of the PROSPER Steering Group representing umbrella networks such as The Survivors Trust; included in professional press communications (eg, Association of Directors of Adult Social Services newsletter) and publicised on the PROSPER website and social media.

**Table 1:** Inclusion and exclusion criteria for staff eligible to complete surveys

VSS providers	Commissioners
Inclusion criteria	Inclusion criteria
Senior staff member who is the nominated representative/lead practitioner from their organisation	Senior commissioner whose role is to commission services from VSS providers for victim-survivors of SV
Currently in post or had been in post within the previous 12 months	Currently in post or had been in post within the previous 12 months
Exclusion criteria	Exclusion criteria
Staff not routinely involved in planning/decision-making about obtaining and managing funding or who are not involved in commissioning processes for their service	Commissioners not routinely involved in planning/decision-making about VSS for victim-survivors of SV

SVsexual violenceVSSvoluntary sector specialist

Surveys were anonymous, and participants were only asked for brief sociodemographic data (age, sex and ethnicity) and some information about their job (eg, role and time in current post). This information allowed duplicate responses from the same organisation to be removed and prevented multiple completion of the survey by the same individual. Non-responders received up to two email/telephone reminders.

### Sample size

There were no formal sample size requirements, and no participant sampling was undertaken, as we aimed to obtain responses from as many organisations as possible. We sought one response per organisation for VSS providers, as surveys focused on respondents’ experiences at the organisational rather than individual level. Duplicate responses from the same organisation were deleted and only the first response received (chronologically) was retained. For commissioners, it was recognised that there may be multiple individuals involved in VSS commissioning with different remits within an organisation (eg, adult vs CYP services). Here, we aimed to obtain as many responses as appropriate from each organisation.

### Data analysis

Data were analysed descriptively using Microsoft Excel, to characterise VSS service provision for SV and commissioning across England. Respondent views about service quality, and how commissioning was perceived to work were also analysed descriptively to identify differences and commonalities by stakeholder group. The small number of responses prohibited subgroup analysis, and missing data could not be imputed. Free-text comments were analysed thematically and are presented throughout the results section alongside the quantitative data rather than separate to it, in order to provide additional context for the quantitative data reported.

### Patient and public involvement

Patient and public involvement (PPI) was central to this study. The PROSPER study team included a PPI co-applicant (DS). Six coresearchers with lived experience of SV who had used specialist SV support services were also recruited to the study team. The PPI coapplicant and coresearchers had input into the design of the surveys, the wording of survey questions and consideration of the time required to complete the surveys and in identifying organisations to whom the surveys should be sent. The PPI coapplicant and two of the coresearchers (LH and LP) contributed to the survey analysis. The PPI coapplicant and coresearchers were all been involved in developing the dissemination strategy for the survey work, including the preparation of conference abstracts and the dissemination of lay summaries to the wider stakeholder groups relevant to this study.

## Results

A total of 54 responses were returned from VSS providers and 34 from commissioners (table 2). Respondents in both groups were most likely to be female, of white ethnicity, and aged over 50. As our approach to survey dissemination was pragmatic and designed to maximise the reach of the surveys in order to maximise participation, response rates cannot be calculated as the number of recipients is unknown.

**Table 2:** Personal and professional characteristics of survey respondents

Characteristic	VSS providers	Commissioners
	**n (%)** *	**n (%)**
All responses	**54**	**34**
Role		
Manager	13 (24.1)	12 (35.3)
Chief executive officer	33 (61.1)	–
Senior practitioner or commissioner	1 (1.9)	7 (20.6)
Policy officer	–	15 (44.1)
Other (eg, trustee, business manager)	4 (7.4)	–
Time in current post		
<12 months	5 (9.3)	2 (5.9)
1–5 years	29 (53.7)	20 (58.8)
6–10 years	11 (20.4)	10 (29.4)
11–15 years	2 (3.7)	2 (5.9)
16–20 years	4 (7.4)	–
21+ years	3 (5.6)	–
Time in specialist services/commissioning		
<12 months	2 (3.7)	2 (5.9)
1–5 years	12 (22.2)	12 (35.3)
6–10 years	18 (33.3)	11 (32.4)
11–15 years	7 (13.0)	7 (20.6)
16–20 years	5 (9.3)	2 (5.9)
21+ years	10 (18.5)	–
Sex		
Male	5 (9.3)	8 (23.5)
Female	49 (90.7)	25 (73.5)
Age group		
18–30 years	0 (0.0)	2 (5.9)
31–40 years	13 (24.1)	4 (11.8)
41–50 years	13 (24.1)	13 (38.2)
51–60 years	22 (40.7)	12 (35.3)
61+ years	6 (11.1)	2 (5.9)
Ethnic group (self-reported)		
White British	40 (74.1)	30 (88.2)
Other ethnicity	13 (24.1)	2 (5.8)

* Percentages may not equal 100 due to missing responses.

VSSvoluntary sector specialist

Professionally, similar proportions of commissioners (64.7%) and providers (62.0%) had spent fewer than 5 years in their current post, but a large proportion of VSS providers had worked in their field for longer than 15 years (40.8%), compared with 26.5% of commissioners. Geographically, among 43 English counties and metropolitan authorities, 28/43 (65%) across all parts of the country including the north, south, midlands and London and including affluent/less affluent areas, urban/rural areas and a range of ethnic diversity were represented in commissioner survey responses and 34/43 (79.1%) in provider responses. A total of 23/43 counties/metropolitan areas (53.5%) returned at least one response from both VSS provider(s) and commissioner (s). The only geographical areas unrepresented in our sample were Cumbria, Berkshire, Surrey and Bedfordshire.

### VSS services offered

Most VSS providers (34/54, 63.0%) offered services to adults and CYP. One-to-one specialist counselling or psychotherapy was offered most frequently (28/54, 51.9%), followed by play therapy (10/54, 18.5%), systemic therapy (8/54, 14.8%), ISVA support (7/54, 13.0%) and therapeutic cognitive behavioural therapy (4/54, 7.4%). ‘Enhanced’ ISVA support (for service users considered to have multiple and/or complex needs) was offered to specific groups by 24/54 providers (44.4%), specifically: CYP (19/24, 79.2%); males (15/24, 62.5%); ethnic minority service users (12/24, 50.0%); adults with learning disabilities (11/24, 45.8%); LGBT+ (lesbian, gay, bisexual and transgender) individuals (10/24, 41.7%); people experiencing domestic violence (9/24, 37.5%); sex workers (8/24; 33.3%) and victim-survivors accessing accident and emergency services (6/24; 25.0%). Additionally, 30/54 (55.6%) offered distinct well-being/holistic services, and 28/54 (51.9%) provided activism-focused support.

### Service funding

VSS providers reported obtaining funding from multiple and varied funding sources. Funding was most likely to come from charitable trusts (45/54, 83.3%) and fundraising (45/54, 83.3%). The Rape and Sexual Abuse Support Fund (commissioned nationally by the Ministry of Justice or via devolved commissioning from the OPCC) provided funding to 42/54 providers (77.8%). Grants from local authorities, NHS England and CCGs (forerunners to ICBs) gave funding to around a third of responding organisations. Most providers reported funding from more than one source (mean 4.8, range 1–9).

A core theme within free-text comments from VSS providers was the perceived need for increased funding for specialist services (n=15). Concerns were raised about providers competing for the same funding; inconsistency and instability of funding sources; financial restrictions on service scope and a lack of funding available for essential specialist services like ISVAs. Funding pressures were reported to cause numerous staffing challenges within services, impairing providers’ ability to recruit and retain specialist team members in order to provide consistent support with sufficient flexibility to meet service users’ needs (n=11). There was also a widely reported perception that funding constraints limited providers’ ability to meet their client needs effectively, with concerns that waiting lists could not meet demand and the frequent need to restrict the duration of support meant that timeliness of service responses could not be guaranteed, particularly for victim-survivors with complex trauma (n=10).

### Service commissioning

**Figure 1:**
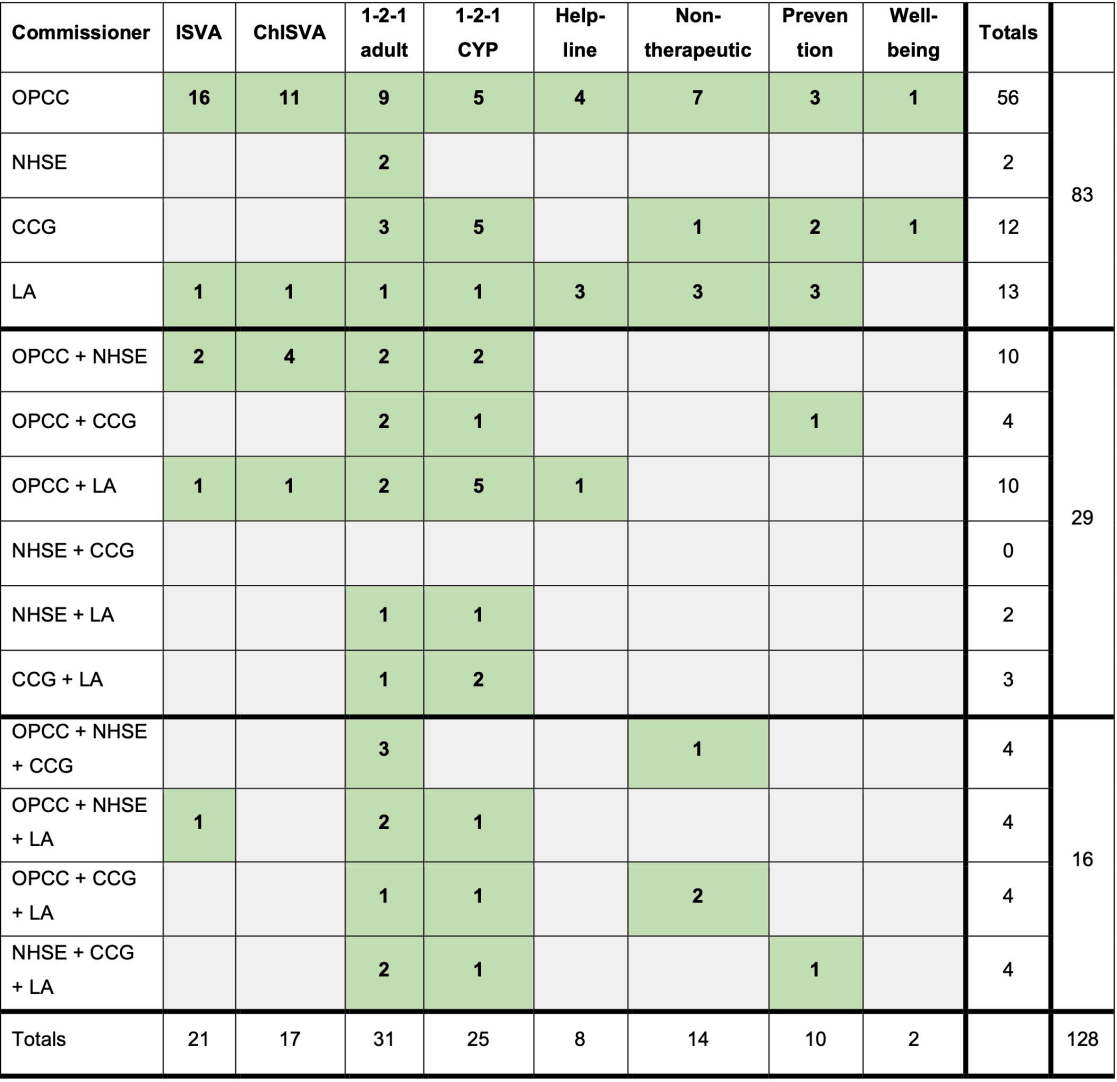
Commissioners of specialist SV services. CCG, Clinical Commissioning Group; ChISVA, Children’s Independent Sexual Violence Advisor; CYP, children and young people; young People; ISVA, Independent Sexual Violence Advisor; LA, local authority; NHS, National Health Service; NHSE, NHS England; OPCC, Office of the Police and Crime Commissioner; SV, sexual violence.

The commissioning landscape was equally complex for VSS providers (figure 1).

A total of 128 commissioned services were described by survey respondents. 83 services (64.8%) were single-commissioned, usually by the OPCC (56/83, 67.5%). 29 services were dual-commissioned (22.7%), typically by the OPCC and NHS England (10/29, 34.5%) or by OPCC and local authorities (10/29, 34.5%) and 16/128 services (12.5%) were triple commissioned. The greatest variety in commissioning in terms of specific service components related to one-to-one adult services (n=31), of which 15 were single commissioned (48.4%), 8 were dual-commissioned (25.8%) and 8 were triple commissioned (25.8%). When asked about their levels of satisfaction with commissioning arrangements for their services, VSS providers were generally satisfied, with 59% of respondents (23/39 services) reporting that they were satisfied or very satisfied, although 12/39 respondents (30.8%) reported ambivalence or dissatisfaction. Rates of satisfaction were highest for engagement with OPCC commissioners, and lowest for CCGs, with 6/17 VSS respondents (35.3%) reporting that they were very dissatisfied with CCG commissioning.

VSS respondents identified numerous barriers and facilitators to effectively working with commissioners. Widely cited positive factors were working with commissioners who understand the SV agenda (96% of respondents), having regular communication with named commissioners (96%) and having good relationships with individual commissioners (95%). The most frequently highlighted barriers were failure to consult with specialist services when developing service specifications (59%), unrealistic timelines for responding to commissioning briefs (59%) and limited opportunities for services to shape service provision (46%). Multiple VSS respondents perceived commissioners to lack awareness of the trauma-informed approach followed by many services (ie, services underpinned by recognising the impact of trauma on individuals and its effect on emotional, social and psychological well-being) and the implications of this for service delivery.

From the commissioner perspective, respondents commended VSS services for their detailed knowledge of SV (91%), the centrality of the victim-survivor voice to service design and delivery (88%) and the holistic approach that many services followed in supporting users’ needs (76%). Nevertheless, commissioners also reported that the evolving structure of commissioning and funding for SV means that services cannot be guaranteed (71%); and that VSS providers may resist providing services to male victim-survivors because services are often underpinned by feminist ideologies (29%), and that services often lack the appropriate infrastructure to collect and process outcomes and monitoring data that commissioners use to plan service provision at the local level (27%).

The impact of austerity measures on funding available for specialist SV services and a complex landscape in which some services were locally commissioned and others commissioned nationally were highlighted in free-text comments by commissioners as being particularly challenging when developing service specifications (n=8). Co-commissioning (when two or more commissioners come together to commission services, either through informal partnership or formal agreements involving pooled budgets) was widely perceived as positive in facilitating improved victim-survivor access to services, particularly for underserved groups (n=14). There was a recognised need for clarity about the operational aspects of co-commissioning, particularly when governance issues arise. A number of commissioner respondents felt that their roles and responsibilities were not set out clearly enough when co-commissioning with others and that there remained a tendency among some service commissioners to pursue a ‘one-size-fits-all’ approach which may not meet the specific needs of those needing specialist SV support (n=9).

### Perceived impacts of changes to funding and commissioning

Table 3 shows comparative data from VSS respondents and commissioners relating to the perceived impacts of changes to funding and commissioning in recent years. VSS providers were most likely to report an increased need for partnership working (61.1%), increased provision of short-term funding (59.3%) and the development of positive relationships with commissioners and other organisations (50.0%). Commissioners also recognised these positive relationships (52.9%) and highlighted an increase in different commissioners working together to commission services (50.0). Again, an increased need for partnership working was highlighted (41.2%). There were notable disparities in views between groups relating to whether or not there had been an increase in short-term funding, with commissioners substantially less likely to report this than VSS providers. Similarly, while 35.2% of providers believed that emotional and practical support was increasingly being prioritised over therapy for victim-survivors, only 5.9% of commissioners perceived this to be the case.

**Table 3:** Perceptions of regional commissioning/funding changes in the last 5 years

Factor	VSS providers agreeing (%)	Commissioners agreeing (%)	Difference in proportions between groups
Increased need for partnership working	61.1	41.2	19.9
Increases in short-term funding	59.3	11.8	47.5
Development of positive relationships	50.0	52.9	2.9
Money brought into the region	40.7	38.2	2.5
Prioritisation of support over therapy	35.2	5.9	29.3
Different commissioners working together	33.3	50.0	17.0
Increase in mid to long-term funding	16.7	29.4	12.7
Money taken out of the region	13.0	2.9	10.1
Closure of specialist SV services	7.4	5.9	1.5

SVsexual violenceVSSvoluntary sector specialist

### Partnership working

A number of VSS providers reported developing partnerships between themselves and statutory services, or with other voluntary service providers. Partnership working was more commonly reported with other voluntary services (n=35, 64.8%) than statutory providers (n=26, 48.1%). Partnerships with statutory services focused largely on the formation of joint referral pathways (n=22, 40.7%). Partnerships with other voluntary organisations were more varied and were most likely to encompass joint referral pathways (n=25, 46.3%), sharing organisational space/resources with other voluntary organisations (n=21, 38.9%) and jointly developed training (n=16, 29.6%). The perception that partnership work needed to improve was frequently cited by commissioner respondents in free-text comments (n=19). Joint working was described as a key means to ensure that services and commissioners could work effectively together, allowing diverse services which were more closely aligned to the needs of local populations to be developed and implemented (n=13).

### Perceived under-representation of specific groups in SV services

Ensuring that victim-survivors from under-represented groups have equity of access to specialist services was a key area for improvement reported by commissioners, and there was a perception in free-text responses that there were multiple barriers to accessing services for some groups (n=11). Many commissioners reported multiple measures taken to improve service provision for under-represented groups, including proactive consultation with services (n=10, 29.4%) and providing funding for outreach/bespoke service development (n=9, 26.5%). Comparative analysis of VSS provider and commissioner perceptions about under-represented groups in specialist SV services (table 4) largely demonstrates concordance in views about the most under-represented victim-survivors, which were felt to be adults and CYP from black and minority ethnic backgrounds.

**Table 4:** Provider and commissioner perceptions about under-represented groups in SV services

Potentially under-represented group	VSS providers agreeing (%)	Commissioners agreeing (%)	Difference in proportions between groups
Adult victim-survivors			
Black and ethnic minority backgrounds	63.0	73.3	10.3
Refugees and asylum seekers	63.0	40.0	23.0
Adults with learning disabilities	39.0	46.7	7.7
Older adults (60+)	37.0	40.0	3.0
Disabled adults	33.3	53.3	20.0
LGBT+ adults	28.0	33.0	5.0
Men	25.9	73.3	47.4
CYP victim-survivors			
Refugees and asylum seekers	50.0	46.7	3.3
CYP from black and ethnic minority backgrounds	48.1	60.0	11.9
Disabled CYP	37.0	40.0	3.0
CYP with learning difficulties	29.6	26.7	2.9
LGBT+CYP	25.9	33.3	7.4
Boys	20.4	53.3	32.9
Children aged <5	13.0	6.7	6.3

CYPchildren and young peopleLGBT+Lesbian, gay, bisexual and transgenderSVsexual violenceVSSvoluntary sector specialist

However, there were substantial disparities in views about the under-representation of disabled adults, refugees/asylum seekers, boys and men. Over half of VSS provider respondents (n=29, 53.7%) reported establishing specific services to engage with under-represented victim-survivors (online supplemental file 5). These included services and support for victim-survivors from multiple ethnic groups, refugees/asylum seekers, mothers, CYP, sex workers, rough sleepers, service users with addictions and trans/non-binary individuals. Support provided to these groups included community engagement activities; support in multiple languages including sign language; social media activities to raise awareness of SV and racism; social groups; specific referral pathways; partnerships with charities, homeless shelters and youth groups, and codevelopment of resources between services and service users from under-represented communities.

## Discussion

To our knowledge, this study is the first to assess VSS service provision and commissioning for SV in England and to take a comparative approach to understanding provider and commissioner perspectives.^36^ As others have found, VSS providers rely on funding from multiple, often short-term sources and in competition with their peers,^14^ and insecure contracts can threaten the independence and sustainability of VSS services.^13 37^ This impacts staffing levels,^38^ providers’ ability to recruit and retain staff and the timeliness and scope of support available.^39^ In turn, this may impact providers’ ability to provide effective and consistent services in their local areas^25^ and a core theme from survey free text comments was the perceived need for increased funding for specialist services to address current barriers to providing consistent, appropriately tailored specialist support for service users, particularly those with complex trauma. Our findings here reflect barriers and concerns about service provision highlighted in other similar studies of SV services within both the statutory and non-statutory sectors.^20 21^

Our results also show extensive co-commissioning of services. While this promotes ‘joined-up’ services, it reflects a trend towards larger contracts that may favour larger providers against which smaller, grassroots providers cannot compete, or it may force smaller providers to change their service scope to meet commissioners’ changing requirements.^15 17^ Co-commissioning and pooled budgets may reduce the funding streams available to services and make services more precarious. This may affect the scope and quality of service provision between areas,^40 41^ and suggests that the effectiveness with which commissioners and providers can work together may depend on the relationships between those involved and shared understanding of each other’s role and expertise.

There was a strong evidence that commissioners recognise the unique contribution that VSS service providers can make to meeting the needs of victim-survivors through delivering support not offered by non-specialist organisations or those within the statutory sector. This was primarily in relation to perceptions about specialist providers’ in-depth knowledge and understanding of SV, the inclusion of the victim-survivor voice in service design/delivery and the holistic approach typically taken to service provision. Commissioners emphasised the value of VSS providers working in partnership with other statutory and voluntary providers to increase service capacity and efficiency. Around half of VSS survey respondents reported partnership working with statutory providers, and around two-thirds with other voluntary organisations. This suggests numerous examples of good relationships between providers, although effective partnerships may be challenging due to competition for obtaining funding and contracts.^25 42^

Survey data provide clear evidence of unmet needs, with both providers and commissioners identifying groups of victim-survivors who remain underserved by specialist services. One finding which would benefit from further study is the perception from some commissioners that male victim-survivors may face difficulties accessing services underpinned by feminist ideologies. Other research has also highlighted the potential impact of the ideological standpoint taken by VSS provider organisations on the scope of the support they provide,^43 44^ although the majority of VSS services work with both women and men. The ongoing under-representation of specific groups within specialist services suggests a need for closer relationships between specialist minoritised services and VSS providers to support cross-referral, which has been recognised by others.^31^ Existing research has highlighted numerous barriers faced by under-represented groups in accessing VSS services for SV, including cultural issues and stigma/taboo; geographical location of services and physical accessibility; language issues; lack of awareness that services exist and the perception among some groups that services are ‘not for them’.^29^,^31^ However, there is also encouraging evidence that both commissioners and providers can add value to core services through the provision of innovative support such as support groups, language and culturally specific support, social media activities, community outreach, awareness raising initiatives and bespoke referral pathways which can help to meet the needs of victim-survivors who may face challenges in accessing services. Indeed, many VSS providers reported that they had developed specific services for under-represented groups such as refugees and asylum seekers, older women, mothers, trans/non-binary individuals, sex workers, the homeless and others.

### Strengths and weaknesses

The number of survey responses was small in comparison to the estimated number of specialist service providers (n=200+) across England. It is not possible to quantify how many commissioning organisations may have some responsibility for SV, although at the time the surveys were administered, there were 41 OPCC organisations and 106 CCGs. The latter were not represented in survey responses at all, despite health services frequently making referrals to VSS services. Additionally, there are over 300 local authorities across England. The small number of responses also meant that our analysis was entirely descriptive and we were unable to undertake any multivariate or subgroup analyses. There may be self-selection bias in our survey responses as participation was voluntary and non-probability (random) sampling of respondents was not possible in the absence of national lists of organisations from which to sample. As a result, we cannot be sure how representative our sample was of the wider VSS or commissioning sector for SV. As our approach was pragmatic and the surveys were disseminated via numerous routes to maximise participation, response rates cannot be calculated as the number of recipients is unknown. However, our study remains one of the few pieces of empirical work designed to focus specifically on VSS service provision and commissioning for SV in England.

### Meaning of the study

Most empirical research in SV has focused on statutory services and the SV voluntary sector has been under-researched. Indeed, the PROSPER study was funded following a nationally commissioned call reflecting NIHR research priorities. This study emphasises the distinctiveness of the SV voluntary sector.^45^ First, there are no statutory duties (only guidance) in relation to support for victim-survivors of SV which differs substantially from other ‘mixed economies’ of welfare such as adult and children’s social care or mental health.^38 46^ This lack of statutory duty contributes to ambiguities about responsibilities for funding and commissioning and what should be considered ‘core’ and ‘specialist services’. This effectively means that the voluntary sector is the only source of support for some groups of victim-survivors.^47 48^ The SV sector has been affected disproportionately by funding cuts, the introduction of new commissioning processes for specialist services and the devolution of service commissioning responsibilities compared with other parts of the voluntary and community sector.^46^ The lack of formal definition of duties means that different commissioners/funders often advocate different approaches, for example, criminal justice versus health responses which can result in services that fail to meet survivors’ needs. Furthermore, the localism of most specialist VSS services means they are particularly vulnerable to changes in funding and contracting requirements, potentially leaving some localities with no specialist provider and the deprioritisation of specific forms of service provision has significant impacts on service users.^5 49 50^ Finally, it has been argued that VSS services in SV face unique challenges in generating income because SV and the shame/blame/trauma associated with it are considered unappealing cases to support. SV does not garner the same sympathy as other social issues, and this has been found to impact directly on services’ ability to raise revenue for SV.^47 48^

### Implications

Our work suggests a need for in-depth analysis of commissioner and provider networks, partnerships and working practices to elucidate the barriers to and facilitators of effective working in localities with differing governance and funding arrangements for SV services. Challenges around partnership working, and the degree to which the voluntary sector engages with statutory services such as SARCs are key avenues for future research in this area.^33 51^ Such research must also engage with a broader range of commissioning organisations such as local authorities and ICBs, where there may be important differences in the nature of services being commissioned and the arrangements for doing so. Qualitative studies are needed to substantiate the implications for practitioners, commissioners and policy-makers and to engage with service users’ views and expectations of VSS services for SV.^52 53^

## Conclusions

These national surveys have provided empirical evidence of a complex, dynamic and evolving funding and commissioning landscape. While there are excellent examples of partnership working and service provision, the surveys highlight pressure in the sector that is exacerbated by funding and commissioning arrangements, with clear evidence of unmet needs among victim-survivors, particularly among minoritised groups.

## supplementary material

10.1136/bmjopen-2024-087810 online supplemental file 1

10.1136/bmjopen-2024-087810 online supplemental file 2

10.1136/bmjopen-2024-087810 online supplemental file 3

10.1136/bmjopen-2024-087810 online supplemental file 4

10.1136/bmjopen-2024-087810 online supplemental file 5

## Data Availability

Data are available on reasonable request.
