# Supplementary material for: Voluntary sector specialist service provision and commissioning for victim-survivors of sexual violence: results from two national surveys in England
Source: BMJ Open. 2024 Sep 13;14(9):e087810. doi: 10.1136/bmjopen-2024-087810 (PMC11407223; doi:10.1136/bmjopen-2024-087810)
Supplement: online supplemental file 3 [file bmjopen-14-9-s003.pdf]

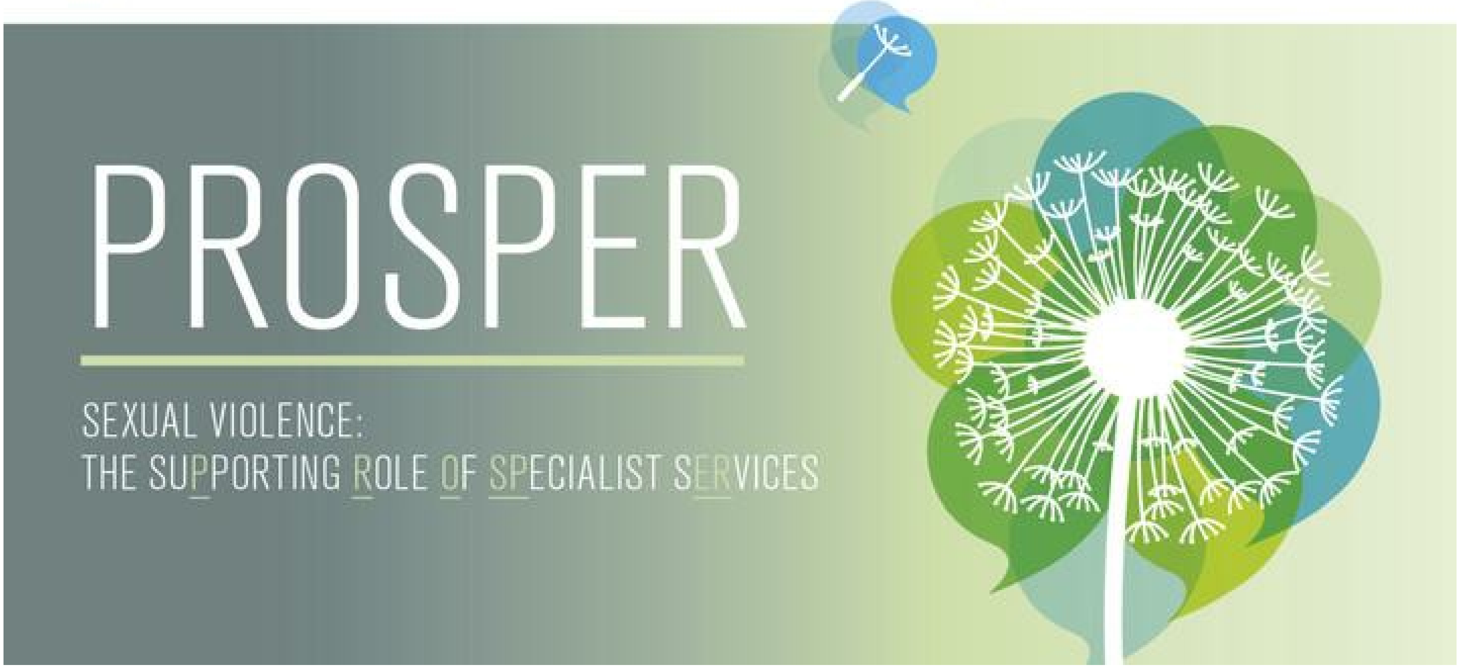

PROSPER Specialist Voluntary Services

Information for participants

About this research

As a specialist provider of voluntary services that are used by victim-survivors of sexual violence, you are invited to participate in a 20 minute online survey, the findings from which will generate evidence about what victim-survivors value and want from services. The survey questions are informed by 30 interviews with practitioners, managers, commissioners and users of voluntary and statutory sector sexual violence services. The survey is being carried out as part of the PROSPER study, which aims to generate new understanding about how specialist services in England, with a focus on those in the voluntary sector, should be commissioned (and funded) to support victim-survivors to thrive in the long term.

Study background

As a result of changes to the structure and funding of health and criminal justice systems in England, the funding and commissioning of services for sexual violence survivors has become

increasingly complex over the past decade. These changes have had a considerable impact on specialist voluntary sector services, who have historically led service innovation and delivery. To date, there is limited academic research about the implications of these funding and commissioning changes on voluntary sector services and, consequently, on the quality and nature of support victim-survivors receive. The PROSPER study will begin to address this gap in the research.

Study details

This in an independent research study, led by Professor Caroline Bradbury-Jones, University of Birmingham, and runs from 1st October 2019 – 31st July 2022. The study is funded by the National Institute for Health Research (NIHR). The study findings, however, will be those of the research team/University of Birmingham and will not necessarily reflect the view of the NIHR or the Department of Health and Social Care.

This study is distinct from the NIHR-funded MESARCH project ('Multidisciplinary Evaluation of Sexual Assault Referral Centres for Better Health'), which you may or may not be familiar with, and which is led by Coventry University. Longer term, these two studies will support a broad understanding of specialist sexual violence provision and result in some joint dissemination activities/outputs.

What do we mean by 'sexual violence' and 'victim-survivor'?

In this survey we use the term ‘sexual violence’ to encompass all forms of sexual violence and abuse experienced by children, young people and adults, including child sexual abuse and sexual exploitation. Whilst recognising the limitations of terminology, we use the term ‘victim-survivor’ to refer to children, young people and adults.

Who should complete this survey?

Our ideal is for only one survey to be completed per organisation and for the person completing it to be someone who leads, works in a senior role or has strategic oversight of the organisation’s operations. If you do not think that person is you, please forward the survey link on to someone you feel is better placed to respond. If you work for a large voluntary sector organisation that provides multiple specialist services in different areas in England (e.g. the NSPCC, Barnardo's), please complete the survey based on the services in your area/region and that relate specifically to sexual violence.

What does participating in this research involve?

- Participation involves completing an online survey comprising closed and free text questions. You can contribute as little or as much detail as you like to those free text

questions.
- If you want to save your responses and complete the survey later, you can do so.
- You will not be asked to give specific information about victim-survivors and we ask that you do not share information which could identify someone.
- The questions principally concern your organisation's work supporting victim-survivors and your views about the funding and commissioning of specialist services. It is not anticipated that the survey will negatively affect you. Should a question touch on issues which you would prefer not to discuss, you do not have to answer that question.

The survey will remain open until 30th June 2021.

Benefits of taking part

You will be contributing to a project that is committed to improving services for victim-survivors of sexual violence in England. All data collected will be used to make recommendations for improving funding and commissioning arrangements and effecting positive change. This is therefore an excellent opportunity to share your perspectives, experiences, frustrations and good practice.

Your rights to confidentiality and anonymity

- Your responses will be kept strictly confidential and we do not ask for identifying information (e.g. your name or date of birth). Whilst we do ask for your job role and who you work for, this will be redacted as soon as the survey closes and never used to identify you.
- Data from the research will be combined in final reports and anonymised excerpts from your survey may be used in publicly accessible reports and articles.
- Anonymised survey responses will be securely shared with members of the research team who are based at the University of Birmingham. The NIHR will not have access to the data you submit.
- Cookies, your IP address and personal data stored by your Web browser are not used in this survey.
- Please be aware that it will not be possible to retract your survey contribution once you have pressed the ‘Finish’ button.
- At the end of the research project your survey response will be destroyed.
- The research team (based at the University of Birmingham) are responsible for the safe collection and storage of your data.

The study has been approved by the University of Birmingham Science, Technology, Engineering and Medical research ethics committee (Ref: ERN_19-1152B). The study is sponsored by University of Birmingham (Ref: RG_19-163).

If you have any questions about the survey, the research more broadly or want to find out more,

please contact Dr Sarah Damery, co-investigator, University of Birmingham: s.l.damery@bham.ac.uk or 0121 414 3343.

Consent

Please read the consent statements below.

- I have read the information about this research and understand what it is about.
- I understand that I have the right to refuse to answer particular questions and that I can end the survey at any time.
- It is clear to me that I cannot ask for my survey response to be removed once I have clicked the 'Finish' button.
- I understand that if any of my quoted words are used in any published document, any identifying details I have disclosed will be changed so that my anonymity will be protected (and the anonymity of any person/organisation to whom I refer).
- I agree to take part in this research project.

1. I consent to participate in this survey. ✱ *Required*

- ○ Yes
- ○ No

Your role and professional background

2. **What is your current role?**

[ ]

2.a. If you selected 'Other', please specify:

[ ]

3. **How long have you been in your CURRENT post?**

- ○ Less than 12 months
- ○ 1-5 years
- ○ 6-10 years
- ○ 11-15 years
- ○ 16-20 years
- ○ 21 or more years

4. **How long have you worked in specialist sexual violence services?**

- ○ Less than 12 months
- ○ 1-5 years
- ○ 6-10 years
- ○ 11-15 years
- ○ 16-20 years
- ○ 21 or more years

For the rest of the survey, the questions relate to your **organisation** (i.e. what it does, how it is funded and commissioned, etc.). Some questions ask for an opinion or judgement. As a senior

representative of your organisation, we ask that you answer these questions on behalf of you and your colleagues, as far as you can.

The term area/region refers to the geographic area where you provide services.

Please remember, if you work for a large voluntary sector organisation e.g. NSPCC, Barnardo's, please answer the following questions based on the specialist services in your area/region and that you provide for children and/or young people affected by sexual violence or abuse.

Some questions about your organisation

5. **What is the name of your organisation?**

6. **Which local authorities does your organisation work with/across?**

7. **Would you describe your organisation as providing support in**

- Urban/metropolitan areas
- Rural areas
- Both

8. **What type of organisation are you?**

- Specialist voluntary sector sexual violence service
- Specialist voluntary sector sexual and domestic violence service
- Voluntary organisation with some specialist sexual violence services (e.g. child sexual abuse)
- Other

8.a. If you selected 'Other', please specify:

9. **How many hours per week is your organisation open?**

- ○ 10 hours or less
- ○ 11-19 hours
- ○ 20-40 hours
- ○ 41+ hours
- ○ 24/7

10. **Please enter the (approximate) number of paid staff working for your organisation**

11. **Please enter the (approximate) number of volunteer staff (including trainee counsellors) working for your organisation**

12. **Which groups does your organisation support?** ✱ *Required*

- ☐ Adults
- ☐ Children and young people

12.a. Please specify the age range of children/young people you support (e.g. 14-17 years):

13. **What key principles underpin service provision in your organisation?** Tick all that apply

- ☐ Feminism and/or critical approaches to patriarchy
- ☐ Trauma-informed
- ☐ Attachment theory
- ☐ Person-centred care
- ☐ Best practice/clinical care guidelines
- ☐ Ecological-systems theory
- ☐ Do not identify with a theory or set of defined principles
- ☐ Other

13.a. If you selected 'Other', please specify:

14. **Are you affiliated with an umbrella body?** Tick all that apply

- ☐ Rape Crisis England and Wales
- ☐ The Survivors' Trust
- ☐ Male Survivors' Partnership
- ☐ Not affiliated
- ☐ Other

14.a. If you selected 'Other', please specify:

15. **Does your organisation work in accordance with a set of national service standards/guidelines?** e.g. as set out by an umbrella body, national membership body etc.

- Yes
- No

16. If you answered 'Yes', what standards do you work towards?

The services you provide

17. **What *therapeutic services* do you provide for ADULTS and for how long?** Please tick all that apply

	We don't provide these services	Up to 6 weeks	Up to 12 weeks	Up to 26 weeks	Other
One-to-one counselling or psychotherapy delivered by a specialist in sexual violence	☐	☐	☐	☐	☐
One-to-one counselling or psychotherapy delivered by someone without a specialism in sexual violence	☐	☐	☐	☐	☐

17.a. If you selected 'Other', please specify duration:

18. **What *therapeutic services* do you provide for CHILDREN AND YOUNG PEOPLE and for how long?** Tick all that apply

	We don't provide these services	Up to 6 weeks	Up to 12 weeks	Up to 26 weeks	Other
One-to-one counselling or psychotherapy delivered by a specialist in sexual violence	☐	☐	☐	☐	☐
One-to-one counselling or psychotherapy delivered by someone without a specialism in sexual violence	☐	☐	☐	☐	☐
Therapeutic CBT	☐	☐	☐	☐	☐

Systemic or multi-systemic therapy (e.g. child and carer, family group work)	☐	☐	☐	☐	☐
Creative or play therapy	☐	☐	☐	☐	☐

18.a. If you selected 'Other', please specify duration:

19. **Do you provide Independent Sexual Violence Advisor (ISVA) services?**

○ Yes ○ No

20. **Who are your ISVA services available to?**

- ○ All victim-survivors
- ○ Only certain groups of victim-survivors

20.a. If only available to certain groups, please specify which:

21. **Do you have additional/unique ISVA services providing support to any of the following groups?** Tick all that apply

- ☐ Children and young people
- ☐ Sex workers
- ☐ Victim-survivors accessing A&E
- ☐ People who identify as LGBT+
- ☐ Male victim-survivors
- ☐ Black and Ethnic Minority (BAME) people
- ☐ People experiencing domestic and sexual violence
- ☐ Adults with learning disabilities
- ☐ Other

21.a. If you selected 'Other', please specify:

22. Do you provide other support services? Tick all that apply

- ☐ We do not provide these services
- ☐ Dedicated support worker providing one-to-one practical and emotional support for adults
- ☐ Dedicated support worker providing one-to-one practical and emotional support for children and young people
- ☐ Social support, peer support and/or self-help groups for victim-survivors
- ☐ Social support, peer support and/or self-help groups for family members of victim-survivors
- ☐ Other

22.a. If you selected 'Other', please specify:

23. If you provide a dedicated helpline for victim-survivors, how many hours does this operate per week?

24. What prevention/education services do you provide? Tick all that apply

- ☐ We do not provide these services
- ☐ Community education (e.g. awareness-raising in schools)
- ☐ Professional training (e.g. to social workers, police)
- ☐ Other

24.a. If you selected 'Other', please specify:

25. **What online/digital support services do you provide?** Tick all that apply

- ☐ We do not provide these services
- ☐ Online video counselling
- ☐ Telephone counselling
- ☐ Text message service support
- ☐ Instant messaging service support (live chat)
- ☐ Email support
- ☐ Other

25.a. If you selected 'Other', please specify:

26. **What wellbeing or holistic health support services do you provide?** Tick all that apply

- ☐ We do not provide these services
- ☐ Yoga
- ☐ Massage
- ☐ Reiki
- ☐ Acupuncture
- ☐ Aromatherapy
- ☐ Sound therapy
- ☐ Hairdressing/beauty treatments

- [ ] Dance and movement therapy
- [ ] Animal-assisted therapy
- [ ] Arts-based approaches (e.g. crafting, painting)
- [ ] Other

26.a. If you selected 'Other', please specify:

27. **Do you carry out activism-focused activities with victim-survivors?** Tick all that apply

- [ ] We do not carry out these activities
- [ ] Support to organise/attend a demonstration or mass meeting
- [ ] Support to engage in online activism
- [ ] Support to celebrate dates of activism and awareness (e.g. White Ribbon)
- [ ] Other

27.a. If you selected 'Other', please specify:

28. **How are victim-survivors involved in the design and delivery of your services?** Tick all that apply

- [ ] Victim-survivors are not involved
- [ ] Via consultation about the introduction of new services

- ☐ Via a victim-survivor steering/advisory group
- ☐ Victim-survivors have written or co-produced service materials
- ☐ Victim-survivors are trained to deliver services as volunteers
- ☐ Victim-survivors are paid members of staff
- ☐ Victim-survivors sit on the board of trustees
- ☐ Other

28.a. If you selected 'Other', please specify:

Criteria for accessing your services

29. **Do you operate a waiting list for any of your therapeutic, support or health services?**

- ○ Yes
- ○ No

30. **Roughly how long do victim-survivors typically have to wait before receiving services?**

31. **Does your organisation signpost or offer support to victim-survivors waiting to access services?** If so, please tell us how - tick all that apply

- ☐ No services available
- ☐ Telephone/helpline support
- ☐ Online/digital support
- ☐ Group work
- ☐ Individual peer support (e.g. mentoring)
- ☐ Wellbeing and welfare services (e.g. relaxation techniques)
- ☐ Signposting to non-specialist support (e.g. Samaritans)
- ☐ Signposting to umbrella organisations (e.g. Survivors' Trust)
- ☐ Other

31.a. If you selected 'Other', please specify:

Commissioning and funding

32. **Are any/all of your sexual violence services currently commissioned?**

- Yes
- No, but previously we have provided commissioned sexual violence services
- No

33. Please tell us who currently commissions which of your sexual violence services.
Tick all that apply

	Office of the Police and Crime Commissioner	NHS England	Clinical Commissioning Group (CCG)	Local Authority	We don't provide this service
Adult Independent Sexual Violence Advisor (ISVA) services - specific qualification	☐	☐	☐	☐	☐
Child Independent Sexual Violence Advisor (ChISVA) services - specific qualification	☐	☐	☐	☐	☐
One-to-one therapeutic services delivered by qualified counsellor or psychotherapist (adult)	☐	☐	☐	☐	☐
One-to-one therapeutic services delivered by qualified counsellor or psychotherapist (child)	☐	☐	☐	☐	☐
Helpline services (e.g. dedicated telephone support)	☐	☐	☐	☐	☐
Non-therapeutic support (e.g. support workers, group work)	☐	☐	☐	☐	☐
Prevention/education services (e.g. training, awareness-raising)	☐	☐	☐	☐	☐
Wellbeing and holistic health services (e.g. massage, yoga)	☐	☐	☐	☐	☐
Other	☐	☐	☐	☐	☐

33.a. If you have other commissioned services, please tell us what they are and who commissions them below:

34. **In your area, are sexual violence services commissioned in the same contract as any other services?**

- No
- Yes, with domestic violence and abuse services
- Yes, with community safety services
- Yes, with children's social care or safeguarding services
- Yes, with other services

34.a. If you selected 'Other services', please specify:

35. **When did the commissioning contract for your sexual violence services start and when does it last until?**

36. **How satisfied are you with the commissioning arrangements for sexual violence services in your area overall, and in relation to each of the commissioning**

groups? We recognise that you may commission with different local authorities and CCG commissioners. Please try and provide a response that reflects a 'general' view about that specific commissioning body.

Please don't select more than 1 answer(s) per row.

	Very satisfied	Satisfied	Neither satisfied nor dissatisfied	Dissatisfied	Very dissatisfied	N/A
Overall	☐	☐	☐	☐	☐	☐
Office for the Police and Crime Commissioner	☐	☐	☐	☐	☐	☐
NHS England	☐	☐	☐	☐	☐	☐
CCG	☐	☐	☐	☐	☐	☐
Local Authority	☐	☐	☐	☐	☐	☐

37. **In your experience, how important have the following been in enabling your organisation to work well with commissioners?** Tick all that apply and tell us if a given activity doesn't happen in your area

Please don't select more than 1 answer(s) per row.

	Very important	Important	Neutral	Slightly important	Not important	This doesn't happen
Regular communication with named commissioners	☐	☐	☐	☐	☐	☐
In-person meetings with commissioners	☐	☐	☐	☐	☐	☐
Working in partnership with other services	☐	☐	☐	☐	☐	☐
Commissioners who understand the sexual violence agenda	☐	☐	☐	☐	☐	☐
Commissioners who understand the voluntary sector	☐	☐	☐	☐	☐	☐
Good relationships with individual commissioners	☐	☐	☐	☐	☐	☐
Consultation during all stages of the commissioning process	☐	☐	☐	☐	☐	☐
Commissioners leading workshops/training programmes about the commissioning process	☐	☐	☐	☐	☐	☐

37.a. If there are any other important factors not listed above, please describe them:

38. **In your experience, which of the following factors have hampered your organisation's ability to work well with commissioners?** Tick all that apply

	This has hampered our ability to work with commissioners
Lack of time to develop good relationships with commissioners	☐
Unrealistic timelines to respond to commissioning briefs/tenders	☐
Failure to consult with specialist voluntary sector services when developing service specifications	☐
Too many commissioners with different agendas	☐
Unrealistic commissioning contracts and expectations	☐
Time-intensive contract management and reporting expectations	☐
Failure to consult with victim-survivors when developing service specifications	☐
Limited ability to shape the terms of service provision	☐

38.a. If there are any other important factors not listed above, please describe them:

39. **Who else currently funds your organisation's services?** Tick all that apply

- ☐ Fundraising and individual/organisational donations
- ☐ Fundraising through fee-paying counselling clients
- ☐ Charitable trusts and foundations (e.g. Lloyds)
- ☐ Charities (e.g. National Lottery)
- ☐ Police and Crime Commissioner grant
- ☐ Clinical Commissioning Group grant
- ☐ Local Authority grant
- ☐ NHS England grant
- ☐ Rape and Sexual Abuse Support Fund (Ministry of Justice)
- ☐ Home Office Violence Against Women and Girls Transformation Fund
- ☐ Tampon Tax Community Fund
- ☐ Other

39.a. If you selected 'Other', please specify:

____

40. **Over the last 5 years, have you seen any of the following in your region as a consequence of commissioning and funding arrangements for sexual violence services?** Tick all that apply

- ☐ Different commissioners working together to fund services
- ☐ Positive relationships between commissioners, funders and specialist sexual violence services
- ☐ More money being brought into the region
- ☐ Money being taken out of the region
- ☐ An increase in short-term funding arrangements
- ☐ An increase in generic and/or private providers winning sexual violence contracts/tenders
- ☐ An increase in mid to long-term funding arrangements
- ☐ An increased need to work in partnership with other organisations/providers

- [ ] Greater competition between specialist voluntary sector services
- [ ] Greater camaraderie between specialist voluntary sector services
- [ ] Commissioners/funders prioritising 'support' work over therapeutic provision
- [ ] Closure of specialist sexual violence services
- [ ] Larger contracts/tenders being used
- [ ] Prioritisation of 'value for money'
- [ ] Other

40.a. If you selected 'Other', please specify:

41. **Over the last 5 years, have any of the following occurred in your organisation as a consequence of commissioning and funding arrangements for sexual violence services?** Tick all that apply

- [ ] Greater seeking of grant/charitable funding rather than commissioned contracts
- [ ] Attempts to diversify funding to not be over-reliant on any one funder
- [ ] The loss of successful sexual violence projects/strands of work
- [ ] Substantial changes or scaling back of projects/strands of work
- [ ] Job cuts or redundancies
- [ ] Staff stress and anxiety
- [ ] Victim-survivor stress and anxiety
- [ ] Opportunity to innovate or re-design services/delivery
- [ ] Opportunity to develop new, positive partnerships with other organisations
- [ ] Feeling forced to work with partners who do not share a similar ethos/approach
- [ ] Opportunities to develop new, positive relationships with commissioners
- [ ] Increased use of paid, short-term staff positions
- [ ] Under-investment in services to specific groups (e.g. BAME, LGBT+)
- [ ] The driving up of service standards and quality

☐ Other

41.a. If you selected 'Other', please specify:

Referral pathways and service linkages

In this section, we ask about: 1) what services your organisation refers victim-survivors to, and 2) areas of innovation in partnership working

42. **Where do you refer or signpost victim-survivors to?** Tick all that apply

	Adults	Children and young people
Other voluntary sector specialist sexual violence services	☐	☐
Voluntary sector specialist domestic abuse services (e.g. Women's Aid)	☐	☐
Sexual Assault Referral Centres (SARCs)	☐	☐
Mental health services	☐	☐
Social services (safeguarding and social care)	☐	☐
Police	☐	☐
General practices	☐	☐
Sexual health services	☐	☐
Other health services (e.g. A&E, health visiting)	☐	☐
Refugee, asylum and immigration support services	☐	☐
Substance misuse services	☐	☐
Housing and homelessness support	☐	☐
Financial/benefits support and advice	☐	☐

42.a. If there are others, please specify below:

42.b. **Which three services do you most frequently refer/signpost victim-survivors to?**

43. **Where do you receive referrals FROM?** Tick all that apply

	Adults	Children and young people
Other voluntary sector specialist sexual violence services	☐	☐
Voluntary sector specialist domestic abuse services (e.g. Women's Aid)	☐	☐
Sexual Assault Referral Centres (SARCs)	☐	☐
Mental health services	☐	☐
Social services (safeguarding and social care)	☐	☐
Police	☐	☐
General practice	☐	☐
Sexual health services	☐	☐
Other health services (e.g. A&E, health visiting)	☐	☐
Refugee, asylum and immigration support services	☐	☐
Substance misuse services	☐	☐
Housing and homelessness support	☐	☐
Financial/benefits support and advice	☐	☐

43.a. If there are others, please specify below:

43.b. **Which three services do you most frequently receive victim-survivor referrals from?**

44. **Approximately what percentage of your referrals are self-referrals?** Please specify (e.g. 50%)

45. **Have you developed any services or partnerships with STATUTORY SECTOR organisations (e.g. Improved Access to Psychological Therapies - IAPT, or Children and Adolescent Mental Health Services - CAMHS)?** Tick all that apply

- ☐ Co-funded worker or service (e.g. ISVA, therapist)
- ☐ Co-located worker or service (i.e. worker from your organisation going to a different service or vice versa)
- ☐ Please tick if the co-located worker or service is integrated and involves joint assessment
- ☐ Joint development or delivery of training (e.g. to practitioners, community groups)
- ☐ Sharing organisational space or resources (e.g. meeting rooms)
- ☐ Referral pathway specifically enabling victim-survivors to access a service
- ☐ We have not developed services or partnerships with statutory organisations
- ☐ Other

45.a. If you selected 'Other', please specify below:

46. **Have you developed any services or partnerships with other VOLUNTARY SECTOR organisations?** Tick all that apply

- ☐ Co-funded worker or service (e.g. ISVA, therapist)
- ☐ Co-located worker or service (i.e. worker from your organisation going to a different service or vice versa)
- ☐ Please tick if the co-located worker or service is integrated and involves joint assessment
- ☐ Joint development or delivery of training (e.g. to practitioners, community groups)
- ☐ Sharing organisational space or resources (e.g. meeting rooms)
- ☐ Referral pathway specifically enabling victim-survivors to access a service
- ☐ We have not developed services or partnerships with voluntary organisations
- ☐ Other

46.a. If you selected 'Other', please specify below:

Different groups of victim-survivors

47. **Are there any ADULT groups which are under-represented within your service?** Tick all that apply

	Under-represented	Not eligible for our service
Black and Minority Ethnic (BAME) adults	☐	☐
Lesbian, Gay, Bisexual and Trans (LGBT+) adults	☐	☐
Adult men	☐	☐
Adult refugees and asylum seekers	☐	☐
Disabled adults	☐	☐
Adults with learning difficulties	☐	☐
Older adults (60+ years)	☐	☐

47.a. If there are any other under-represented groups, please specify below:

48. **Are there any groups of CHILDREN AND YOUNG PEOPLE which are under-represented in your service?** Tick all that apply

	Under-represented	Not eligible for our service
BAME children and young people	☐	☐
LGBT+ children and young people	☐	☐
Boys and young men	☐	☐
Children/young people who are refugees or asylum seekers	☐	☐
Disabled children and young people	☐	☐

Children and young people with learning difficulties	☐	☐
Children under 5 years	☐	☐

48.a. If there are any other under-represented groups, please specify below:

49. **Please tell us about any services your organisation has developed to engage with under-represented groups** (e.g. a refugee and migrant counselling service, older adults' support group)

Capturing victim-survivors' journeys

When we talk about outcomes, we mean the results, benefits and changes arising from the use of your services

50. **Do you use outcome measures to monitor victim-survivors' progress when using your organisation's services?** ✱ *Required*

- ○ Yes
- ○ No

51. **What informs decisions about the outcome measures you collect?** Tick all that apply

- ☐ National standards/guidelines of a governing body
- ☐ The requirements of commissioners and/or funders
- ☐ Professional expertise and judgement
- ☐ Other

51.a. If you selected 'Other', please specify:

52. **Which of the following victim-survivor outcomes do you monitor?** Tick all that apply

	Adults	Children and young people
Feelings of depression	☐	☐
Phobic experiences	☐	☐
Obsessive/compulsive behaviours (OCD)	☐	☐
Post-Traumatic Stress Disorder (PTSD) symptoms	☐	☐
Physical health (e.g. sleep, headaches, eating)	☐	☐
Work/educational engagement	☐	☐
Social activities (e.g. going to the cinema, reading)	☐	☐
Intimate and family relationships	☐	☐
Self-esteem	☐	☐
Feelings of autonomy	☐	☐
Sense of safety	☐	☐
Confidence	☐	☐
Re-integration into community/sense of belonging	☐	☐
Knowledge of accessing support services	☐	☐
No longer being in an abusive household	☐	☐

52.a. If your organisation monitors other outcomes, please describe them:

53. **How frequently do you collect outcomes data from victim-survivors?**

- At the start and end of support
- At the start, middle and end of support
- On an ad hoc basis
- Other

53.a. If you selected 'Other', please specify:

Victim-survivor feedback

54. **HOW do you collect feedback from victim-survivors on the nature and quality of service they have received from your organisation?** Tick all that apply

- ☐ We do not collect feedback
- ☐ Informally (e.g. conversations during sessions)
- ☐ Through use of a specific feedback form/checklist
- ☐ Through use of complaints procedures and monitoring
- ☐ Other

54.a. If you selected 'Other', please specify:

55. **WHEN do you collect feedback from victim-survivors who use your services?**

- ○ We do not collect feedback
- ○ At regular points throughout their service use
- ○ In their final session with the service
- ○ Follow-up contact after they have finished using the service
- ○ Other

55.a. If you selected 'Other', please specify:

56. **Do you collect feedback from victim-survivors who exit services early?**

- ○ We do not attempt to contact these victim-survivors
- ○ We do attempt to contact them

57. **If you do attempt to contact them, please describe how:**

Some information about you

58. **Please describe your gender identity**

59. **Have you ever identified as a trans person?**

60. **What is your age group?**

61. **How would you describe your ethnicity?**

Reflections/concluding thoughts

We recognise that voluntary sector specialist services operate in a complex and sometimes challenging environment that includes limited funding and fragmentation between services

62. **With the above in mind, how well do you feel that your organisation meets the needs of victim-survivors?**

Please don't select more than 1 answer(s) per row.

	Very well	Well	Acceptably	Poorly	Very poorly
Extent that victim-survivors' needs are met	☐	☐	☐	☐	☐

63. **Are there any areas of your organisation's provision and/or partnerships with other services that you feel need to be improved?**

64. **If there is anything you would like to add about any of the questions in the survey, or something you feel we have not covered, please tell us below:**

Final page

Thank you for taking the time to participate in this survey – your response has now been submitted.

Just to remind you, if you would like to be kept informed of project progress or if you have questions about the research, please contact Dr Sarah Damery, Co-investigator, at s.l.damery@bham.ac.uk or 0121 414 3343.

The findings from our study will be posted on our project website so please look here for information and updates (https://www.birmingham.ac.uk/research/applied-health/research/PROSPER-study.aspx). You will be able to access information there or by emailing Dr Sarah Damery directly.

If you have any concerns about ethical issues and/or the conduct of the research, please contact Dr Birgit Whitman, Head of Research Governance & Integrity at b.whitman@bham.ac.uk (0121 415 8011). Dr Whitman does not work as part of the research team and has no involvement in the study.

Key for selection options

2 - What is your current role?

Chief Executive Officer (CEO)
Service Manager
Lead practitioner
Trustee
Other

23 - If you provide a dedicated helpline for victim-survivors, how many hours does this operate per week?

We do not provide this
10 hours or less
11-19 hours
20-40 hours
41+ hours

58 - Please describe your gender identity

Female
Male
Non-binary

Prefer not to say

59 - Have you ever identified as a trans person?

Yes

No

Prefer not to say

60 - What is your age group?

18-30

31-40

41-50

51-60

61+
