# Supplementary material for: Voluntary sector specialist service provision and commissioning for victim-survivors of sexual violence: results from two national surveys in England
Source: BMJ Open. 2024 Sep 13;14(9):e087810. doi: 10.1136/bmjopen-2024-087810 (PMC11407223; doi:10.1136/bmjopen-2024-087810)
Supplement: online supplemental file 4 [file bmjopen-14-9-s004.pdf]

UNIVERSITY OF BIRMINGHAM | RISK, ABUSE AND VIOLENCE (RAV) RESEARCH PROGRAMME

FUNDED BY NIHR | National Institute for Health Research

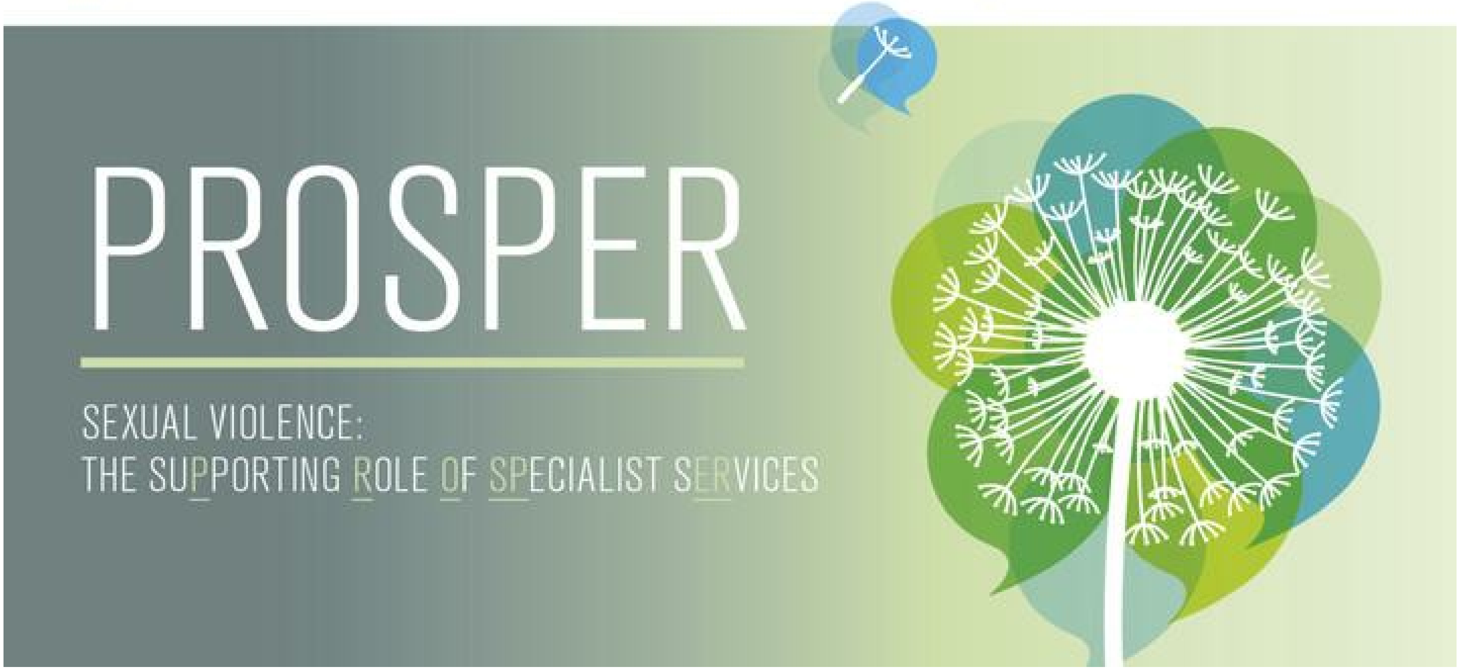

PROSPER Commissioners

Information for participants

About this research

As a commissioner of services that are used by adult and/or child victim-survivors of sexual violence, you are invited to participate in a 20 minute online survey, the findings from which will generate evidence about what victim-survivors value and want from services. They will also inform the development of commissioning guidelines, to help bring greater clarity and consistency to thearea of specialist sexual violence service commissioning. The survey questions are informed by 30 interviews with practitioners, managers, commissioners and users of voluntary and statutory sector sexual violence services. The survey is being carried out as part of the PROSPER study, which aims to generate new understanding about how specialist services in England, with a focus on those in the voluntary sector, should be commissioned (and funded) to support victim-survivors to thrive in the long term.

Study background

As a result of changes to the structure and funding of health and criminal justice systems in England, the funding and commissioning of services for sexual violence survivors has become increasingly complex over the past decade. To date, there is limited academic research about the implications of these funding and commissioning changes on service providers and, consequently, on the quality and nature of support victim-survivors receive. The PROSPER study will begin to address this gap in the research.

Study details

This in an independent research study, led by Professor Caroline Bradbury-Jones, University of Birmingham, and runs from 1st October 2019 – 31st July 2022. The study is funded by the National Institute for Health Research (NIHR). The study findings, however, will be those of the research team/University of Birmingham and will not necessarily reflect the view of the NIHR or the Department of Health and Social Care.

This study is distinct from the NIHR-funded MESARCH project ('Multidisciplinary Evaluation of Sexual Assault Referral Centres for Better Health'), which you may be familiar with, and which is led by Coventry University. Longer term, these two studies will support a broad understanding of specialist sexual violence provision and result in some joint dissemination activities/outputs.

What do we mean by 'sexual violence' and 'victim-survivor'?

In this survey we use the term 'sexual violence' to encompass all forms of sexual violence and abuse experienced by children, young people and adults, including child sexual abuse and sexual exploitation. Whilst recognising the limitations of terminology, we use the term 'victim-survivor' to refer to children, young people and adults.

Who should complete this survey?

You should complete this survey if you are a commissioner for NHS England, an Office for the Police and Crime Commissioner, a Clinical Commissioning Group (CCG) or a local authority **and** your role involves the commissioning/joint-commissioning of services for/ that are used by sexual violence victim-survivors. This specifically means if you commission/joint-commission:

- **Specialist services whose primary purpose is to support sexual violence victim-survivors** e.g. voluntary sector Rape Crisis Centres, Survivors' Trust affiliated organisations, statutory/private Sexual Assault Referral Centres and voluntary sector children's services delivering specialist strands of sexual violence/abuse work.
- **NHS psychological therapies** (e.g. counselling, Cognitive Behavioural Therapy (CBT), Eye Movement Desensitisation Reprocessing (EMDR)) or **Tier 3 Child and Adolescent**

Mental Health Services (CAMHS), that are used by sexual violence victim-survivors, even though not typically designed to exclusively support them.

What does participating in this research involve?

- Participation involves completing an online survey comprising closed and free text questions. You can contribute as little or as much detail as you like to those free text questions.
- If you want to save your responses and complete the survey later, you can do so.
- You will not be asked to give specific information about victim-survivors and we ask that you do not share information which could identify someone.
- The questions principally concern your organisation's work commissioning services for victim-survivors and your organisation's views about the voluntary sector in providing specialist services. It is not anticipated that the survey will negatively affect you. Should a question touch on issues which you would prefer not to discuss, you do not have to answer that question.

The survey will remain open until 30th June 2021.

Benefits of taking part

You will be contributing to a project that is committed to improving services for victim-survivors of sexual violence in England. All data collected will be used to make recommendations for improving funding and commissioning arrangements and effecting positive change. This is therefore an excellent opportunity to share your perspectives, experiences, frustrations and good practice.

How will we use information about you?

- We do not ask for any identifying information (e.g. name or date of birth) in this research. Whilst we do ask for your job role and who you commission for, this will be redacted as soon as the survey closes and never used to identify you.
- Any responses you give to the survey will be kept safely and securely.
- Your completed survey will be assigned a code number and you will not be identifiable from your response.
- Data from the research will be combined in final reports and anonymised excerpts from your survey may be used in publicly accessible reports and articles.
- Anonymised survey responses will be securely shared with members of the research team who are based at the University of Birmingham. The NIHR will not have access to the data you submit.
- Cookies, your IP address and personal data stored by your Web browser are not used in

this survey.
- At the end of the research project your survey response will be destroyed.
- The research team (based at the University of Birmingham) are responsible for the safe collection and storage of your data.

The study has been approved by the University of Birmingham Science, Technology, Engineering and Medical research ethics committee (Ref: ERN_19-1152B) and the Health Research Authority (Ref: IRAS 271280; version 2.0, 04/01/2021). The study is sponsored by University of Birmingham (Ref: RG_19-163).

If you have any questions about the survey, the research more broadly or want to find out more, please contact Dr Sarah Damery, co-investigator, University of Birmingham: s.l.damery@bham.ac.uk or 0121 414 3343.

What are your choices about how your information is used?

It will not be possible to retract your survey contribution once you have pressed the 'Finish' button as we will not be able to identify you to remove your data.

Where can you find out more about how your information is used?

You can find out more about how your information will be used by contacting Dr Sarah Damery, co-investigator, University of Birmingham: s.l.damery@bham.ac.uk, or by emailing the University of Birmingham's Data Protection Office at: dataprotection@contacts.bham.ac.uk.

Consent

Please read the consent statements below.

- I have read the information about this research and understand what it is about.
- I understand that I have the right to refuse to answer particular questions and that I can end the survey at any time.
- It is clear to me that I cannot ask for my survey response to be removed once I have clicked the survey 'Finish' button.
- I understand that if any of my quoted words are used in any published document, any identifying details I have disclosed will be changed so that my anonymity will be protected (and the anonymity of any person/organisation to whom I refer).
- I agree to take part in this research project.

1. I consent to participate in this survey. ✱ *Required*

- ○ Yes
- ○ No

Your professional background and commissioning role

2. What is your current job role? ✱ *Required*

3. Who do you commission for? ✱ *Required*

- A Clinical Commissioning Group (CCG)
- A local authority (including Public Health)
- An Office of the Police and Crime Commissioner (OPCC)
- NHS England, Health and Justice
- Other

3.a. If you selected 'Other', please specify:

4. How long have you been in your current commissioning post?

- Less than 12 months
- 1-5 years
- 6-10 years
- 11-15 years
- 16-20 years
- 21 or more years

5. How long have you been a commissioner?

- ◯ Less than 12 months
- ◯ 1-5 years
- ◯ 6-10 years
- ◯ 11-15 years
- ◯ 16-20 years
- ◯ 21 or more years

6. What was your role before you entered commissioning?

7. Please specify (approximately) what percentage of your role involves the commissioning and contract management of services for/that are used by sexual violence victim-survivors?

8. Which key documents influence your work commissioning services for/that are used by victim-survivors of sexual violence? Tick all that apply

- ☐ None
- ☐ Home Office (2019) Commissioning Framework for all commissioners of support services for victims and survivors of child sexual abuse in England
- ☐ Home Office (2016) Violence against Women and Girls Services: supporting local commissioning
- ☐ NHS England (2018) Sexual Assault and Abuse Strategy (SAAS)

- [ ] NHS England (2015) Commissioning Framework: for adult and paediatric Sexual Assault Referral Centres (SARC) services
- [ ] Women's Aid and Imkaan Capacity Building Partnership (2018) Successful Commissioning: a guide for commissioning services that support women and children survivors of violence
- [ ] NWG Network (2018) Child Sexual Exploitation Commissioning Guidance
- [ ] NHS (2019) Long Term Plan
- [ ] Other

8.a. If you selected 'Other', please specify:

9. **What data do you use to inform your broad understanding of sexual violence in the geographic area you work?** Tick all that apply

- [ ] Local police reported data
- [ ] Local sexual violence agency data
- [ ] Local health data
- [ ] Local social care data
- [ ] Local needs assessment
- [ ] Crime survey for England and Wales data
- [ ] National police reported data
- [ ] Academic research
- [ ] Other

9.a. If you selected 'Other', please specify:

Services you commission

To reiterate, by 'commission', we mean if you are a lead or co-commissioner of services.

10. **Across which local authorities do you commission services for/that are used by sexual violence victim-survivors?**

11. **Who do you commission services for?** Tick all that apply

- [ ] Adults
- [ ] Children and young people

11.a. Please specify the age range of child/young person you support (e.g. 14-17 years):

12. **Do you commission *specialist sexual violence services* (e.g. voluntary sector Rape Crisis Centres, Survivors' Trust affiliated organisations, statutory/private Sexual Assault Referral Centres and voluntary sector children's services delivering specialist strands of sexual violence/abuse work)?** ✱ *Required*

- ( ) Yes
- ( ) No

13. Please identify which specialist service(s) you commission for victim-survivors of sexual violence. Tick all that apply

	For adults	For children and young people
Forensic medical services	☐	☐
Sexual health services	☐	☐
Management and crisis response in the forensic setting	☐	☐
Independent Sexual Violence Advisor (ISVA) services - specific qualification	☐	☐
One-to-one therapeutic services delivered by a qualified counsellor or psychotherapist	☐	☐
Practical and emotional support (e.g. dedicated support workers, group work)	☐	☐
Prevention/education services (e.g. training, awareness-raising activities)	☐	☐
Well-being and holistic health services (e.g. massage, creative therapies)	☐	☐
Other	☐	☐

13.a. If you selected 'Other', please specify:

14. Please tell us how satisfied you are with the commissioning arrangements for specialist sexual violence services in your geographical area of responsibility: overall and in relation to the contribution of each of the commissioning groups. We recognise that you may commission these services across/with different local authorities and CCGs. Please try and provide a response that reflects a 'general' view about that commissioning group.

Please don't select more than 1 answer(s) per row.

	Very satisfied	Satisfied	Neither satisfied nor dissatisfied	Dissatisfied	Very dissatisfied	N/A
Overall	☐	☐	☐	☐	☐	☐
Office for the Police and Crime Commissioner	☐	☐	☐	☐	☐	☐
NHS England	☐	☐	☐	☐	☐	☐
CCGs	☐	☐	☐	☐	☐	☐
Local authority	☐	☐	☐	☐	☐	☐
Other body	☐	☐	☐	☐	☐	☐

14.a. If you rated an 'Other body', please tell us which one:

15. **If you commission *NHS psychological therapies* or *Tier 3 CAMHS*, do you commission specific approaches or ways of working with victim-survivors of sexual violence?** Tick all that apply. If you do not commission NHS psychological therapies or Tier 3 CAMHS services, please go to the next question.

	Psychological therapies	Tier 3 CAMHS	We don't commission these approaches/services
A commissioned referral pathway for victim-survivors into these services	☐	☐	☐
A commissioned, bespoke response for victim-survivors within these services (e.g. family therapy for children affected by sexual abuse, psychological therapy specifically designed for victim-survivors)	☐	☐	☐
Other approach	☐	☐	☐

15.a. If you selected an 'Other approach', please describe below:

Developing and awarding contracts

16. **Which of the following do you do when developing specifications for services for/that are used by sexual violence victim-survivors?** Tick all that apply

	Developing specialist sexual violence services	Developing NHS psychological therapies/CAMHS services
Consult with victim-survivors	☐	☐
Consult with local statutory sector specialist sexual violence services (e.g. SARC)	☐	☐
Consult with local voluntary sector specialist sexual violence services (e.g. Rape Crisis)	☐	☐
Consult with non-voluntary sector specialist organisations (e.g. Samaritans)	☐	☐
Consult with the Police about sexual violence	☐	☐
Consult with health services about sexual violence	☐	☐
Consult with social services (adult and/or children) about sexual violence	☐	☐
Use demographics of the population to estimate levels of sexual violence service need	☐	☐
Map sexual violence provision to identify gaps and understand what's needed by who and when	☐	☐
Map current spend on specialist sexual violence provision and response to it across public services	☐	☐
Use evidence of need drawn from national documents (e.g. HMIC reports on rape attrition)	☐	☐
Other	☐	☐

16.a. If you selected 'Other' please explain below

To reiterate, in this survey, specialist services encompass voluntary sector services (e.g. voluntary sector Rape Crisis Centres, Survivors' Trust affiliated organisations, statutory/private Sexual Assault Referral Centres and voluntary sector children's services delivering specialist strands of sexual violence/abuse work)

17. If you commission specialist sexual violence services, what are the *three* most significant factors in determining the award of a contract? Please tick three

Please select no more than 3 answer(s).

- ☐ I do not commission specialist sexual violence services
- ☐ The provider's ability to work in partnership and/or within a consortia arrangement
- ☐ The provider's ability to deliver an integrated service
- ☐ Value for money
- ☐ Wider social benefit or value (social return on investment)
- ☐ The provider's ability to deliver a high quality service
- ☐ The provider's established reputation as a specialist service
- ☐ Evidence of victim-survivor involvement in service design and delivery
- ☐ Other

17.a. If you selected 'Other', please specify:

Voluntary sector specialist sexual violence services

This section is about your views and experiences of **voluntary sector specialist sexual violence services only** (e.g. voluntary sector Rape Crisis Centres, Survivors' Trust affiliated organisations, statutory/private Sexual Assault Referral Centres and voluntary sector children's services delivering specialist strands of sexual violence/abuse work).

Please complete this section even if you don't currently commission voluntary sector specialist services.

18. **What do you think are the strengths and/or unique features of voluntary sector specialist services?** Tick all that apply

- ☐ They offer independence and the ability to critique statutory services
- ☐ Their historic and ongoing role in campaigning for change/victim-survivor voice
- ☐ Their detailed knowledge of sexual violence
- ☐ Their situating of sexual violence within a context of gender inequality
- ☐ Their commitment and capacity to make a victim-survivor feel believed
- ☐ Their holistic approach to assessing and supporting victim-survivors' needs
- ☐ They are less bureaucratic, enabling them to be more innovative
- ☐ They have greater knowledge of and links with their local communities
- ☐ They offer a welcoming environment to victim-survivors
- ☐ Victim-survivor voice is central to service design and delivery
- ☐ They don't have any particular strengths and unique features
- ☐ Unsure
- ☐ Other

18.a. If you selected 'Other', please specify:

19. **What do you think are the limitations of voluntary sector specialist services?** Tick all that apply

- ☐ They are resistant to change e.g. working with new partners
- ☐ They are challenging/have a reputation for being challenging to work with
- ☐ Their historic/current resistance to providing services to male victim-survivors
- ☐ Short term funding/commissioning arrangements mean services cannot be guaranteed
- ☐ Staff don't have sufficient training/expertise to support individuals with complex needs
- ☐ They don't have the technological and administrative infrastructure to collect and process outcomes/monitoring data
- ☐ They don't provide anything distinct to equivalent therapeutic services in the statutory sector
- ☐ There are no limitations to these services
- ☐ Unsure
- ☐ Other

19.a. If you selected 'Other', please specify:

20. **As a commissioner, how do you financially support voluntary sector specialist services?** Tick all that apply

- ☐ We do not financially support these services
- ☐ Competitive tendering/commissioning
- ☐ Grants
- ☐ Unsure
- ☐ Other

20.a. If you selected 'Other', please specify:

21. **Do you currently commission any voluntary sector specialist sexual violence services?** ✱ *Required*

- Yes
- No

22. **Please tell us which voluntary sector specialist sexual violence services you commission.** Tick all that apply

	For adults	For children and young people
ISVA services (specific qualification)	☐	☐
One-to-one therapeutic services delivered by a qualified counsellor or psychotherapist	☐	☐
Helpline services (i.e. dedicated telephone support)	☐	☐
Emotional and practical support (e.g. group work, family support)	☐	☐
Prevention/education services (i.e. training, awareness raising)	☐	☐
Well-being and holistic health services (e.g. massage, creative therapies)	☐	☐
Other	☐	☐

22.a. If you selected 'Other', please describe below:

23. **As a commissioner, have you been involved in any of the following activities to support voluntary sector specialist sexual violence services engage in the commissioning process?** Tick all that apply

☐ Providing training and/or upskilling support about the commissioning process

☐ Making monies available for piloting and seed project work to create partnerships and joint working arrangements

☐ Providing consultation/discussion opportunities with your organisation

☐ We haven't been involved in engagement work of this nature with voluntary sector services

☐ Other

23.a. If you selected 'Other', please specify:

24. **In your view, what is the optimum commissioning contract length for the delivery of voluntary sector sexual violence services?**

25. **Are you currently able to award contracts for this length of time?**

- Yes
- No

25.a. Please specify why not:

26. **If you do not commission any voluntary sector specialist sexual violence services, please tell us why not.** Tick all that apply

- ☐ Not applicable - I do commission voluntary sector specialist services
- ☐ They don't offer anything distinct to existing services within the statutory or private sectors
- ☐ There aren't any high quality voluntary sector specialist services in the area that I commission
- ☐ I don't fully understand what voluntary sector specialist services do
- ☐ Too often, voluntary sector specialist services only support women and girls
- ☐ There is no evidence base that highlights the value or impact of voluntary sector specialist services
- ☐ I would like to commission voluntary sector specialist services but don't have the funding to allocate
- ☐ Other

26.a. If you selected 'Other', please specify:

Outcomes and monitoring data

27. **Are you regularly provided with monitoring data for victim-survivors broken down by key demographics?** Please tick all that apply

- ☐ No, I don't see demographic data
- ☐ Yes, by gender identity
- ☐ Yes, by sexual orientation
- ☐ Yes, by race
- ☐ Yes, by disability
- ☐ Yes, by age
- ☐ Yes, by religion or belief

28. **If you commission NHS psychological therapy services, are you regularly provided with data that indicates whether service users are victim-survivors of sexual violence?**

- ○ I don't commission mental health services
- ○ No - I commission these services but don't see this data
- ○ Yes - I commission these services and see this data

29. **The next sections of the survey are about the commissioning/co-commissioning of SARCs and voluntary sector specialist sexual violence services only. If you do not commission these services, please answer 'no' below, to skip to the end of the survey. If you do commission/co-commission these services, please tick 'yes'** ✱ *Required*

- ○ Yes
- ○ No

Collecting and responding to outcomes and monitoring data

30. **Do you commission SARC services?** ✱ *Required*

- Yes
- No

31. When capturing the results, benefits and changes (i.e. outcomes) arising from victim-survivors' use of SARC SERVICES, what do you think are the three most important outcomes to monitor?

Please select no more than 3 answer(s).

- ☐ Feelings of depression
- ☐ Phobic experiences
- ☐ Obsessive/compulsive behaviours (OCD)
- ☐ Post-Traumatic Stress Disorder (PTSD) symptoms
- ☐ Physical health (e.g. sleep, eating)
- ☐ Work/educational engagement
- ☐ Social activities (e.g. going to the cinema, reading)
- ☐ Intimate and family relationships
- ☐ Self-esteem
- ☐ Feelings of autonomy
- ☐ Sense of safety
- ☐ Confidence
- ☐ Re-integration into community/sense of belonging
- ☐ Knowledge of accessing support services
- ☐ No longer being in an abusive household
- ☐ Other

31.a. If you selected 'Other', please specify:

32. If you commission SARC services for ADULTS, which (if any) of the following groups of adult victim-survivors are under-represented in the SARC services you commission? Tick all that apply. If you do not commission adult SARC services, please go to the next question.

	Under-represented	Not sure if under-represented
Black and Minority Ethnic (BAME) adults	☐	☐
Lesbian, Gay, Bisexual and Trans (LGBT+) adults	☐	☐
Men	☐	☐
Refugees and asylum seekers	☐	☐
Disabled adults	☐	☐
Adults with learning difficulties	☐	☐
Older adults (60+ years)	☐	☐

32.a. If there are any other under-represented groups, please describe them below

33. **If you commission SARC services for CHILDREN AND YOUNG VICTIM-SURVIVORS of sexual violence, which (if any) of the following groups are under-represented in the SARC services you commission?** Tick all that apply. If you do not commission SARC services for children and young people, please go to the next question.

	Under-represented	Not sure if under-represented
BAME children and young people	☐	☐
LGBT+ children and young people	☐	☐
Boys and young men	☐	☐
Children/young people who are refugees or asylum seekers	☐	☐
Disabled children and young people	☐	☐
Children and young people with learning difficulties	☐	☐
Children under 5	☐	☐

33.a. If there are any other under-represented groups, please describe them below

IF YOU *DO NOT* COMMISSION VOLUNTARY SECTOR SPECIALIST SERVICES, PLEASE PROCEED TO QUESTION 37

34. **When capturing the results, benefits and changes (i.e. outcomes) arising from victim-survivors' use of VOLUNTARY SECTOR specialist services, what do you think are the three most important outcomes to monitor?**

Please select no more than 3 answer(s).

- [ ] Feelings of depression
- [ ] Phobic experiences
- [ ] Obsessive/compulsive behaviours (OCD)
- [ ] Post-Traumatic Stress Disorder (PTSD) symptoms
- [ ] Physical health (e.g. sleep, eating)
- [ ] Work/educational engagement
- [ ] Social activities (e.g. going to the cinema, reading)
- [ ] Intimate and family relationships
- [ ] Self-esteem
- [ ] Feelings of autonomy
- [ ] Sense of safety
- [ ] Confidence
- [ ] Re-integration into community/sense of belonging
- [ ] Knowledge of accessing support services
- [ ] No longer being in an abusive household
- [ ] Other

34.a. If you selected 'Other', please specify:

35. **If you commission ADULT voluntary sector specialist sexual violence services,**

which (if any) of the following groups of adult victim-survivors are under-represented in the voluntary sector specialist services you commission? Tick all that apply. If you do not commission these services for adults, please go to the next question.

	Under-represented	Not sure if under-represented
Black and Minority Ethnic (BAME) adults	☐	☐
Lesbian, Gay, Bisexual and Trans (LGBT+) adults	☐	☐
Men	☐	☐
Refugees and asylum seekers	☐	☐
Disabled adults	☐	☐
Adults with learning difficulties	☐	☐
Older adults (60+ years)	☐	☐

35.a. If there are other under-represented groups, please describe them below

36. **If you commission voluntary sector specialist sexual violence services for CHILDREN AND YOUNG PEOPLE, which (if any) of the following groups of children and young victim-survivors are under-represented in the voluntary sector specialist services you commission?** Tick all that apply. If you do not commission these services for children and young people, please go to the next question.

	Under-represented	Not sure if under-represented
BAME children and young people	☐	☐
LGBT+ children and young people	☐	☐
Boys and young men	☐	☐
Children/young people who are refugees or asylum seekers	☐	☐

Disabled children and young people	☐	☐
Children and young people with learning difficulties	☐	☐
Children under 5	☐	☐

36.a. If there are other under-represented groups, please describe them below

37. **Thinking about the SARC and/or specialist voluntary sector sexual violence services you commission, are there any measures you have taken to try and improve the provision of specialist services for victim-survivors from under-represented groups?** Tick all that apply.

- [ ] As commissioners, we consult with specialist services who work with/champion under-represented groups (e.g. BAME people) when developing service specifications
- [ ] We set explicit standards around, and monitor, levels of service satisfaction amongst individuals from under-represented groups
- [ ] We set explicit standards around, and monitor, a provider's outreach activity to promote their service to under-represented groups
- [ ] We set explicit standards around, and monitor, a provider's engagement in consultation and/or shared decision-making with individuals from under-represented groups
- [ ] We have made funding available to carry out outreach with and/or provide bespoke services to victim-survivors from under-represented groups
- [ ] Other

37.a. If you selected 'Other', please specify:

# Joint commissioning

38. **Do you joint commission any specialist sexual violence services?** ✱ *Required*

- ◯ Yes
- ◯ No

39. **Who do you joint commission with?** Tick all that apply

- ☐ NHS England
- ☐ CCG
- ☐ Local authority
- ☐ Office of the Police and Crime Commissioner
- ☐ Other

39.a. If you selected 'Other', please specify:

40. **If joint commissioning involves a pooled/combined budget, do you contribute to that budget?**

- ○ No - there is no pooled budget
- ○ No - we do not contribute to the pooled budget
- ○ Yes - we contribute to the pooled budget

41. **If you do not joint commission specialist sexual violence services, what factors have prevented it?** Tick all that apply

- ☐ It's too logistically complex
- ☐ Concern over different commissioners' priorities and agendas
- ☐ Not enough time to develop partnerships with other commissioners
- ☐ No clear commissioning/strategic lead to drive a joint agenda
- ☐ It's not necessary to meet our remit/obligations
- ☐ We plan to co-commission in the future
- ☐ Other

41.a. If you selected Other, please specify:

Reflections on commissioning specialist sexual violence services

42. **In your experience, how important have the following been in enabling your organisation to commission specialist services for victim-survivors of sexual violence?** Tick all that apply and tell us if a given activity doesn't happen in your area

Please don't select more than 1 answer(s) per row.

	Very important	Important	Neutral	Slightly important	Not important	This doesn't happen
National service standards	☐	☐	☐	☐	☐	☐
Regular communication with named individuals in specialist provider organisations	☐	☐	☐	☐	☐	☐
Regular, face-to-face meetings with providers	☐	☐	☐	☐	☐	☐
Consulting with specialist sexual violence services at each stage of the commissioning process	☐	☐	☐	☐	☐	☐
Offering training and/or workshop activities to support providers' ability to engage in the commissioning process	☐	☐	☐	☐	☐	☐

Engaging with victim-survivors at each stage of the commissioning process	☐	☐	☐	☐	☐	☐

42.a. If there are any other important factors, please list them here:

43. **In your experience, which of the following factors have hampered your organisation's ability to commission specialist services for victim-survivors of sexual violence?** Tick all that apply

- ☐ Lack of time to develop good relationship with specialist providers
- ☐ Having to work across different local authority areas
- ☐ Having to work across different CCG areas
- ☐ Potential clashes between health and criminal justice priorities
- ☐ Too many commissioners with different agendas
- ☐ Lack of current funding available
- ☐ Lack of time to complete various elements of the commissioning process
- ☐ The impact of austerity measures over the last 10 years
- ☐ Being unable to access comprehensive data about rates of sexual violence in my area
- ☐ Providers taking a gender specific approach to the delivery of services (i.e. not supporting men)
- ☐ Certain aspects of sexual violence service provision still being commissioned at the national level, despite the push for local commissioning approaches
- ☐ Lack of evidence about the comparative effectiveness of approaches/ interventions in the field of sexual violence
- ☐ Sub-contracting arrangements
- ☐ Other

43.a. If you selected 'Other', please specify:

44. **In your geographical area of responsibility, over the LAST 5 YEARS, have you seen any of the following happen as a consequence of commissioning and funding arrangements for specialist sexual violence services?** Tick all that apply

- ☐ Different commissioners working together to fund services
- ☐ Positive relationships between commissioners, funders and specialist services
- ☐ Driving up of service standards
- ☐ More money being brought into the region
- ☐ Money being taken out of the region
- ☐ An increase in short-term (2 years or less) commissioned contracts
- ☐ An increase in mid to long-term (4 years or more) commissioned contracts
- ☐ Providers working in partnership with other organisations
- ☐ Commissioners prioritising 'support' work over therapeutic provision
- ☐ Greater competition between voluntary sector services
- ☐ Greater seeking of grant/ charitable funding by SARCs
- ☐ Under-investment in the development of services to specific groups (e.g. BAME, LGBT+ etc.)
- ☐ Closure of specialist services
- ☐ A reduction in service standards
- ☐ Other

44.a. If you selected 'Other', please specify:

Some information about you

45. **Please describe your gender identity**

46. **Have you ever identified as a trans person?**

47. **What is your age group?**

48. **How would you describe your ethnicity?**

Reflections/concluding thoughts

49. Overall, how well do you feel that the services you commission meet the needs of victim-survivors of sexual violence?

Please don't select more than 1 answer(s) per row.

	Very well	Well	Neither well nor poorly	Poorly	Very poorly	N/A
Voluntary sector specialist services	☐	☐	☐	☐	☐	☐
SARCs	☐	☐	☐	☐	☐	☐
Psychological therapies services	☐	☐	☐	☐	☐	☐
Tier 3 CAMHS	☐	☐	☐	☐	☐	☐

50. **Are there any areas of your organisation's commissioning work or its partnerships with other commissioners that you feel need to improve? If so, please tell us more:**

51. **If there is anything you would like to add about any of the questions in the survey, or something you feel we have not covered, please tell us below:**

Final page

Thank you for taking the time to participate in this survey – your response has now been submitted.

Just to remind you, if you would like to be kept informed of project progress or if you have questions about the research, please contact Dr Sarah Damery, Co-investigator, at s.l.damery@bham.ac.uk or 0121 414 3343.

The findings from our study will be posted on our project website so please look here for information and updates (https://www.birmingham.ac.uk/research/applied-health/research/PROSPER-study.aspx). You will be able to access information there or by emailing Dr Sarah Damery directly.

If you have any concerns about ethical issues and/or the conduct of the research, please contact Dr Birgit Whitman, Head of Research Governance & Integrity at b.whitman@bham.ac.uk (0121 415 8011). Dr Whitman does not work as part of the research team and has no involvement in the study.

Key for selection options

7 - Please specify (approximately) what percentage of your role involves the commissioning and contract management of services for/that are used by sexual violence victim-survivors?

Less than 10%
Between 10-30%
Between 30-50%
Between 50-75%
More than 75%

45 - Please describe your gender identity

Female
Male
Non-binary
Prefer not to say

46 - Have you ever identified as a trans person?

Yes
No
Prefer not to say

47 - What is your age group?

18-30
31-40
41-50
51-60
61+
