# Supplementary material for: Voluntary sector specialist service provision and commissioning for victim-survivors of sexual violence: results from two national surveys in England
Source: BMJ Open. 2024 Sep 13;14(9):e087810. doi: 10.1136/bmjopen-2024-087810 (PMC11407223; doi:10.1136/bmjopen-2024-087810)
Supplement: online supplemental file 5 [file bmjopen-14-9-s005.pdf]

ADDITIONAL FILE 1: Voluntary sector specialist services developed to engage with under-represented victim-survivor groups

Groups	Services
Victim-survivors from specific ethnic groups	• Outreach service and community engagement ISVA<br>• Foreign language counsellors to engage with non-native English speakers<br>• Post to facilitate ethnic group outreach and education; new referrals pathway<br>• Community ISVAs; counselling in a range of locally-spoken languages<br>• Using social media to raise awareness and dispel myths<br>• ISVA for people who have experienced racism<br>• Asian women's group in partnership with community organisation<br>• Chinese women's social group<br>• Specialist counselling for South Asian women
Refugees and asylum seekers	• Partnership with the county-wide refugee and asylum service<br>• New pathways into specialist refugee and asylum seeker organisations<br>• Refugee counselling/refugee support worker<br>• Refugee women's social group<br>• Links with local migrant support organisation
Older women	• Targeted services for older women<br>• Project for over 55s to tackle under-representation amongst this group<br>• Counselling, support work, training, resources for older women
Mothers	• Development of a mother's support group
Children and young people	• Children's ISVA (13 years and under)<br>• Partnership with Barnardo's<br>• Specialist young women and young men counsellors<br>• Work with local youth agencies
Trans/non-binary	• Support group established
Complex needs	• Multiple and complex needs ISVA
Learning disabilities (LD)	• LD and autism service (counselling, support work, training and development)<br>• Specialist LD counsellor<br>• Working with local LD organisations
LGBT+	• LGBT+ ISVA
Sex workers	• Sex worker ISVA
Men	• Working with male support charities to increase access and referrals<br>• Therapy for male victim-survivors;
Homeless	• Working with homeless shelters to increase referrals
Service users with addictions	• Online dance based groupwork; volunteer peer support programme for victim-survivors with addictions
Students	• Ambassador project to engage with university community
Disabilities	• Co-development of a video raising awareness of information and community support to respond to deaf victim-survivors with British Sign Language as first language; community workshops for deaf communities

Groups	Services
General activity to increase engagement	• Specialist ISVAs; Engagement ISVAs<br>• Funding for travel expenses and childcare for service users<br>• Informal partnership with specialist organisations for under-represented groups<br>• Development of leaflets and materials<br>• Dedicated outreach to increase engagement from 'easy to ignore' groups<br>• Action plans to address under-representation<br>• Partnership working with organisations who support under-represented groups

ISVA=Independent Sexual Violence Advisor; LGBT+=Lesbian, Gay, Bisexual, Transgender+
