# Supplementary material for: Voluntary sector specialist service provision and commissioning for victim-survivors of sexual violence: results from two national surveys in England
Source: BMJ Open. 2024 Sep 13;14(9):e087810. doi: 10.1136/bmjopen-2024-087810 (PMC11407223; doi:10.1136/bmjopen-2024-087810)
Supplement: Supplementary file 6 [file bmjopen-2024-087810.reviewer_comments.pdf]

PEER REVIEW HISTORY

BMJ Open publishes all reviews undertaken for accepted manuscripts. Reviewers are asked to complete a checklist review form and are provided with free text boxes to elaborate on their assessment. These free text comments are reproduced below.

ARTICLE DETAILS

**TITLE (PROVISIONAL)**	Voluntary sector specialist service provision and commissioning for victim-survivors of sexual violence: Results from two national surveys in England
**AUTHORS**	Damery, Sarah; Gunby, Clare; Hebberts, Lucy; Patterson, Laura; Smailes, Harriet; Harlock, Jenny; Isham, Louise; Maxted, Fay; Schaub, Jason; Smith, Deb; Taylor, Julie; Bradbury-Jones, C

VERSION 1 - REVIEW

**REVIEWER NAME**	***Widanaralalage , B Kennath***
**REVIEWER AFFILIATION**	King's College London
**REVIEWER CONFLICT OF INTEREST**	Na
**DATE REVIEW RETURNED**	22-May-2024

GENERAL COMMENTS	The manuscript titled "Voluntary sector specialist service provision and commissioning for victim-survivors of sexual violence: Results from two national surveys in England" presents a well-structured and clear overview of voluntary sector services (VSS) and their support provision for victims of sexual violence. However, the introduction could benefit from a more comprehensive discussion of the challenges encountered in providing support to victims of sexual violence. Some of our recent works on SARC Widanaralalage et al., 2024) and other sources from Horvath et al. (2021) highlight several barriers related to underfunding and austerity measures, which should be incorporated into the introduction to provide a more comprehensive context.<br><br>Horvath M, Massey K, Dalton T, Rose H, Matthews K, Crivatu I. Independent Sexual Violence Advisers (ISVAs) in England, Wales and Northern Ireland. https://mdx.figshare.com/articles/online_resource/ISVA_Survey_Report_May_2021_cc-by-nc_pdf/14566638<br><br>Widanaralalage, B.K., Murphy, A.D. & Loughlin, C. Support or justice: a triangulated multi-focal view of sexual assault victim support in a UK sexual assault referral centre (SARC). Int J Ment Health Syst 18, 15 (2024). https://doi.org/10.1186/s13033-024-00631-z<br><br>The methods and results sections are clearly presented, and the authors should be commended for their clarity.<br><br>The discussion section could be improved by commenting in more detail on the findings, particularly the insightful free-text comments from participants. For instance, the comments on page 12, line 243 (onwards) are remarkably similar to the findings from Widanaralalage et al. (2024) mentioned above, where SARC staff members describe various barriers and concerns related to service provision. Highlighting these similarities and the implications of lack of funding would be beneficial for readers, as it demonstrates the widespread nature of such concerns. Moreover, it would help position the submitted manuscript within this growing area of study.<br><br>Another notable finding from the free-text comments is on line 295 (p.13), which suggests that some VSS providers may resist providing services to male victim-survivors due to feminist ideologies underpinning their services (29%). This is an intriguing notion, particularly in light of some of our research with male victims and service providers of sexual violence, which highlights significant barriers to support from "female-focused" services. To further support and position the findings within the existing male rape literature, the following sources could be referenced:<br><br>1. Widanaralalage, B. K., Hine, B. A., Murphy, A. D., & Murji, K. (2022). "I Didn't Feel I Was A Victim": A Phenomenological Analysis of the Experiences of Male-on-male Survivors of Rape and Sexual Abuse. Victims & Offenders, 0(0), 1–26. https://doi.org/10.1080/15564886.2022.2069898<br><br>2. Widanaralalage, B., Hine, B., Murphy, A., & Murji, K. (2023). A Qualitative Investigation of Service Providers' Experiences Supporting Raped and Sexually Abused Men. Violence and Victims, 38, VV–2022. https://doi.org/10.1891/VV-2022-0084<br><br>Finally, a more detailed discussion of the barriers for underrepresented groups highlighted in the results would significantly strengthen the manuscript.<br><br>Overall, the manuscript presents valuable insights into the provision and commissioning of VSS for victim-survivors of sexual violence. By addressing the suggested areas for improvement, particularly in

**REVIEWER NAME**	***Bunce, Annie***
**REVIEWER AFFILIATION**	City University of London, Violence & Society Centre
**REVIEWER CONFLICT OF INTEREST**	Na
**DATE REVIEW RETURNED**	05-Jun-2024

GENERAL COMMENTS

This is my favourite article I have reviewed for a while, so thank you firstly for starting my morning off well. This is a much needed, well written and comprehensive piece of research which takes stock of the current provision and commissioning landscape for specialist sexual violence support services. This paper and the evidence it provides will be useful for service providers, commissioners, policymakers and researchers with an interest in meeting the needs of survivors of sexual violence. I am very happy to reccomend this paper for publication, although I have a couple of suggestions which may further strengthen the paper and a couple of minor grammatical points which I have detailed in a separate document. Thank you for the opportunity to review this very important contribution to the field.

General points

- In some places the points made in the Discussion don't clearly link to findings presented in the Results section- I found myself flicking back and forth between the two sections to piece the narrative together.
    - Line 384 "There was strong evidence that commissioners recognise the unique contribution that VSS service providers can make to meeting the needs of victim-survivors through delivering support not offered by other organisations"- does this refer to the finding on commissioner's perspectives of the holistic approach taken by services (line 293), or the improved service provision for under-represented groups (lines 348-351). Also, who are the 'other organisations' referred to here- statutory/non-specialist services?
    - Line 396 "However, there is also encouraging evidence that both commissioners and providers can add value to core services and meet the needs of victim survivors who may face challenges in accessing services"- again, it would be helpful to more specifically refer back to the results here. How/where do they add value to core services? I think the second part in terms of meeting needs for those who may face challenges accessing services would be addressed by including some of the information from additional file 1 in the main body (see comment below).
- In the data analysis section it says free text comments were thematically analysed and presented alongside quantitative data for context (lines 196-199). Whilst comments from the survey are referred to throughout the results section, might it be possible to include some quotes? This could add to the persuasiveness of the evidence provided and make for an even more interesting read.

Specific points

- Line 92- closing """ missing after the word "consent" and before the citation.
- Line 186- "for commissioner", an "s" missing from the end of the word "commissioner"
- Line 226- "Extra ISVA support"- a definition of what is meant by 'extra' ISVA support (i.e. on top of 'standard' ISVA support) [illegible]

VERSION 1 – AUTHOR RESPONSE

REVIEWER 1

1. The manuscript titled "Voluntary sector specialist service provision and commissioning for victim-survivors of sexual violence: Results from two national surveys in England" presents a well-structured and clear overview of voluntary sector services (VSS) and their support provision for victims of sexual violence. However, the introduction could benefit from a more comprehensive discussion of the challenges encountered in providing support to victims of sexual violence. Some of our recent works on SARC Widanaralalage et al., 2024) and other sources from Horvath et al. (2021) highlight several barriers related to underfunding and austerity measures, which should be incorporated into the introduction to provide a more comprehensive context.

Horvath M, Massey K, Dalton T, Rose H, Matthews K, Crivatu I. Independent Sexual Violence Advisers (ISVAs) in England, Wales and Northern Ireland.
https://mdx.figshare.com/articles/online_resource/ISVA_Survey_Report_May_2021_cc-by-nc_pdf/14566638

Widanaralalage, B.K., Murphy, A.D. & Loughlin, C. Support or justice: a triangulated multi-focal view of sexual assault victim support in a UK sexual assault referral centre (SARC). Int J Ment Health Syst 18, 15 (2024). https://doi.org/10.1186/s13033-024-00631-z

Response: Many thanks for suggesting these additional references and for providing the full citations. We now cite both of these in our introduction (page 5, paragraph beginning 'despite their importance…' which now provides more context about the barriers related to underfunding and austerity measures (and have renumbered the references throughout accordingly).

2. The methods and results sections are clearly presented, and the authors should be commended for their clarity.

Response: Many thanks for your positive view of the clarity of our work.

3. The discussion section could be improved by commenting in more detail on the findings, particularly the insightful free-text comments from participants. For instance, the comments on page 12, line 243 (onwards) are remarkably similar to the findings from Widanaralalage et al. (2024) mentioned above, where SARC staff members describe various barriers and concerns related to service provision. Highlighting these similarities and the implications of lack of funding would be beneficial for readers, as it demonstrates the widespread nature of such concerns. Moreover, it would help position the submitted manuscript within this growing area of study.

Response: We have added a couple of sentences to the paragraph mentioned by the reviewer to reference their work and highlight some of the similarities in findings (page 17, paragraph 1, beginning 'to our knowledge, this study is the first to assess…'). We feel that we highlight the implications of underfunding within the sector extensively within this paragraph already, but now also highlight similarities between these findings and other work in the field, as suggested by the reviewer.

4. Another notable finding from the free-text comments is on line 295 (p.13), which suggests that some VSS providers may resist providing services to male victim-survivors due to feminist ideologies

underpinning their services (29%). This is an intriguing notion, particularly in light of some of our research with male victims and service providers of sexual violence, which highlights significant barriers to support from "female-focused" services. To further support and position the findings within the existing male rape literature, the following sources could be referenced:

1. Widanaralalage, B. K., Hine, B. A., Murphy, A. D., & Murji, K. (2022). "I Didn't Feel I Was A Victim": A Phenomenological Analysis of the Experiences of Male-on-male Survivors of Rape and Sexual Abuse. Victims & Offenders, 0(0), 1–26. https://doi.org/10.1080/15564886.2022.2069898

2. Widanaralalage, B., Hine, B., Murphy, A., & Murji, K. (2023). A Qualitative Investigation of Service Providers' Experiences Supporting Raped and Sexually Abused Men. Violence and Victims, 38, VV–2022. https://doi.org/10.1891/VV-2022-0084

Response: Thank you for this. We do not want to overstate this point, interesting though it is, because the issue was mentioned by a relatively small proportion of commissioners (29%) in comparison to other issues mentioned by far greater numbers of respondents. This was also a perception by commissioners rather than necessarily reflecting what services actually do. However, we have added some text to highlight the issue and now reference both citations suggested by the reviewer as these are important works in a literature that our paper does not really touch upon, so thank you for drawing them to our attention (changes made to page 18, paragraph beginning ‘survey data provide clear evidence of…’).

5. Finally, a more detailed discussion of the barriers for underrepresented groups highlighted in the results would significantly strengthen the manuscript.

Response: Thank you for this point. We have added a brief summary of barriers that under-represented groups may encounter when trying to access services (page 18, paragraph beginning ‘survey data provide clear evidence of…’). We have kept this relatively brief because our response to reviewer 2 also entailed adding more detail about under-represented groups but focused more towards some of the ways that innovative services have overcome barriers rather than focusing purely on challenges. We feel that these changes improve the manuscript with regard to its coverage of under-representation in services.

6. Overall, the manuscript presents valuable insights into the provision and commissioning of VSS for victim-survivors of sexual violence. By addressing the suggested areas for improvement, particularly in the introduction and discussion sections, the authors can enhance the manuscript's contribution to this important field of study.

Response: Many thanks for your positive view of our paper.

REVIEWER 2

1. This is my favourite article I have reviewed for a while, so thank you firstly for starting my morning off well. This is a much needed, well written and comprehensive piece of research which takes stock of the current provision and commissioning landscape for specialist sexual violence support services. This paper and the evidence it provides will be useful for service providers, commissioners, policymakers and researchers with an interest in meeting the needs of survivors of sexual violence. I am very happy to recommend this paper for publication, although I have a couple of suggestions which may further strengthen the paper and a couple of minor grammatical points which I have detailed in a separate document. Thank you for the opportunity to review this very important contribution to the field.

Response: Thank you for your positive view of our work and we are glad that you feel it provides

important and useful evidence for multiple stakeholder groups.

2. In some places the points made in the Discussion don't clearly link to findings presented in the Results section- I found myself flicking back and forth between the two sections to piece the narrative together.
Line 384 "There was strong evidence that commissioners recognise the unique contribution that VSS service providers can make to meeting the needs of victim-survivors through delivering support not offered by other organisations"- does this refer to the finding on commissioner's perspectives of the holistic approach taken by services (line 293), or the improved service provision for under-represented groups (lines 348-351). Also, who are the 'other organisations' referred to here- statutory/non-specialist services?

Response: Thank you for raising this point and apologies for confusion. It was essentially a general point that covered commissioners' positive views about providers' knowledge of SV, the centrality of the victim-survivor voice in service design and delivery, and the holistic approach to service provision. The 'other organisations' referred to are indeed non-specialist services or those from the statutory sector. We have edited the text in the paragraph referenced (page 17, paragraph 2) to make these points clear.

3. Line 396 "However, there is also encouraging evidence that both commissioners and providers can add value to core services and meet the needs of victim survivors who may face challenges in accessing services"- again, it would be helpful to more specifically refer back to the results here. How/where do they add value to core services? I think the second part in terms of meeting needs for those who may face challenges accessing services would be addressed by including some of the information from additional file 1 in the main body (see comment below).

Response: Thank you for this point. We have clarified in that paragraph (second paragraph of discussion, pages 17-18) some of the ways that VSS services add value to core services through innovative service provision, referring back to specific examples of such activities now described in the methods (taken from the supplementary table). We also describe again some of the groups to whom such innovative services are available. We believe that this makes a stronger link back to the results to carry the narrative through to the discussion.

4. In the data analysis section it says free text comments were thematically analysed and presented alongside quantitative data for context (lines 196-199). Whilst comments from the survey are referred to throughout the results section, might it be possible to include some quotes? This could add to the persuasiveness of the evidence provided and make for an even more interesting read.

Response: Thank you for this point. If the 'qualitative' data had come from interviews, it would have been possible to use quotations to illustrate the themes arising. However, the nature of free text comments made by survey respondents does not lend itself to being quoted. Respondents in nearly all cases gave comments in a fragmentary way, almost as bullet points, without sentences e.g. 'not enough money' or 'can't retain good staff' or 'not enough joint working' or 'challenging to establish partnerships.' Whilst these points were insightful and we were able to analyse these comments to derive themes to provide context to the provider and commissioner perspectives established from the quantitative data, we do not feel that it would add to the paper to attempt to include quotations as in the vast majority of cases there was no quotable text to include in the way that would have been the case from interview data. Hence our decision to provide thematic summaries only.

Specific points from reviewer 2:

5. Line 92- closing "”" missing after the word "consent" and before the citation.

Response: This has been corrected.

6. Line 186- “for commissioner”, an “s” missing from the end of the word “commissioner”

Response: This has been corrected.

7. Line 226- “Extra ISVA support”- a definition of what is meant by ‘extra’ ISVA support (i.e. on top of ‘standard’ ISVA support) would be useful here.

Response: Thank you for this point. The term ‘extra’ ISVA support is probably not correct – it would be more correct to call this ‘enhanced’ ISVA support which is typically provided for those considered to have multiple and/or complex needs. We have now corrected this.

8. Lines 356-360- on the specific services to engage under-represented victim-survivors. I think this is one of the most important findings in terms of evidencing some of the innovative work being undertaken, and it’s a shame to have it all in a supplementary file that not everybody will refer to. If word count allows, would it be possible to include a couple of examples of the actual services being offered (in addition to the examples already provided of the groups these services are tailored to). I think this would mean that the sentence from the abstract on the evidence for innovative practice (lines 54-56) would be more strongly demonstrated.

Response: Thank you for this point. We agree. Some examples taken from supplementary file 1 (which has now become supplementary file 3) have been added to the paragraph to exemplify some of the innovative work being done by services to support under-represented groups.

9. Line 448- “such SARCs”- missing the word “as” in the middle.

Response: This has been corrected.

VERSION 2 – REVIEW

**REVIEWER NAME**	***Widanaralalage , B Kennath***
**REVIEWER AFFILIATION**	King's College London
**REVIEWER CONFLICT OF INTEREST**	Na
**DATE REVIEW RETURNED**	26-Jul-2024

**GENERAL COMMENTS**	Thank you for taking our comments and feedback on board. The manuscript is now ready for publication.

**REVIEWER NAME**	***Bunce, Annie***
**REVIEWER AFFILIATION**	City University of London, Violence & Society Centre
**REVIEWER CONFLICT OF INTEREST**	Na

**DATE REVIEW RETURNED**	01-Aug-2024

**GENERAL COMMENTS**	Thank you again for the opportunity to review this paper, and for responding so positively to feedback. The revisions made are excellent and I think this paper is going to be an extremely valuable contribution to the field.
